# Inhibition of Escherichia coli Lipoprotein Diacylglyceryl Transferase Is Insensitive to Resistance Caused by Deletion of Braun’s Lipoprotein

**DOI:** 10.1128/JB.00149-21

**Published:** 2021-06-08

**Authors:** Jingyu Diao, Rie Komura, Tatsuya Sano, Homer Pantua, Kelly M. Storek, Hiroko Inaba, Haruhiko Ogawa, Cameron L. Noland, Yutian Peng, Susan L. Gloor, Donghong Yan, Jing Kang, Anand Kumar Katakam, Michael Volny, Peter Liu, Nicholas N. Nickerson, Wendy Sandoval, Cary D. Austin, Jeremy Murray, Steven T. Rutherford, Mike Reichelt, Yiming Xu, Min Xu, Hayato Yanagida, Junichi Nishikawa, Patrick C. Reid, Christian N. Cunningham, Sharookh B. Kapadia

**Affiliations:** a Department of Infectious Diseases, Genentech, South San Francisco, California, USA; b Peptidream Inc., Kawasaki, Kanagawa, Japan; c Department of Structural Biology, Genentech, South San Francisco, California, USA; d Department of Biochemical and Cellular Pharmacology, Genentech, South San Francisco, California, USA; e Department of Translational Immunology, Genentech, South San Francisco, California, USA; f Department of Pathology, Genentech, South San Francisco, California, USA; g Department of Protein Chemistry, Genentech, South San Francisco, California, USA; h Department of Early Discovery Biochemistry, Genentech, South San Francisco, California, USA; Brigham and Women's Hospital/Harvard Medical School

**Keywords:** Lgt, Lpp, antibiotic resistance, lipoproteins

## Abstract

Lipoprotein diacylglyceryl transferase (Lgt) catalyzes the first step in the biogenesis of Gram-negative bacterial lipoproteins which play crucial roles in bacterial growth and pathogenesis. We demonstrate that Lgt depletion in a clinical uropathogenic Escherichia coli strain leads to permeabilization of the outer membrane and increased sensitivity to serum killing and antibiotics. Importantly, we identify G2824 as the first-described Lgt inhibitor that potently inhibits Lgt biochemical activity *in vitro* and is bactericidal against wild-type Acinetobacter baumannii and E. coli strains. While deletion of a gene encoding a major outer membrane lipoprotein, *lpp*, leads to rescue of bacterial growth after genetic depletion or pharmacologic inhibition of the downstream type II signal peptidase, LspA, no such rescue of growth is detected after Lgt depletion or treatment with G2824. Inhibition of Lgt does not lead to significant accumulation of peptidoglycan-linked Lpp in the inner membrane. Our data validate Lgt as a novel antibacterial target and suggest that, unlike downstream steps in lipoprotein biosynthesis and transport, inhibition of Lgt may not be sensitive to one of the most common resistance mechanisms that invalidate inhibitors of bacterial lipoprotein biosynthesis and transport.

**IMPORTANCE** As the emerging threat of multidrug-resistant (MDR) bacteria continues to increase, no new classes of antibiotics have been discovered in the last 50 years. While previous attempts to inhibit the lipoprotein biosynthetic (LspA) or transport (LolCDE) pathways have been made, most efforts have been hindered by the emergence of a common mechanism leading to resistance, namely, the deletion of the gene encoding a major Gram-negative outer membrane lipoprotein *lpp*. Our unexpected finding that inhibition of Lgt is not susceptible to *lpp* deletion-mediated resistance uncovers the complexity of bacterial lipoprotein biogenesis and the corresponding enzymes involved in this essential outer membrane biogenesis pathway and potentially points to new antibacterial targets in this pathway.

## INTRODUCTION

The cell envelope of a typical Gram-negative bacterium consists of two membranes, namely, a phospholipid inner membrane (IM) and an asymmetrical outer membrane (OM), the latter of which is composed of a phospholipid inner leaflet and a lipopolysaccharide (LPS) outer leaflet. The IM and OM are separated by the periplasm, which contains a peptidoglycan (PGN) cell wall (reviewed in detail in reference 1). Escherichia coli harbors >90 lipoproteins, many of which are localized to the inner leaflet of the OM but can also be exposed on the bacterial cell surface (2, 3). Bacterial lipoproteins play critical roles in adhesion, nutrient uptake, antibiotic resistance, virulence, invasion, and immune evasion (4), making the lipoprotein biosynthetic and transport pathways attractive targets for novel antibacterial drug discovery.

**FIG 1:**
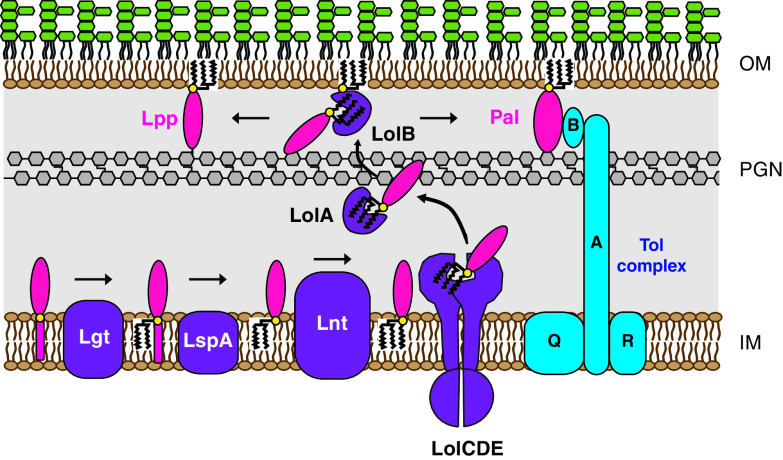
Lipoprotein biosynthesis and transport in Gram-negative bacteria. Prolipoprotein substrates translocate through the IM via the Sec or Tat pathway and are sequentially modified by Lgt, LspA, and Lnt. Triacylated lipoproteins that are destined for the OM are recognized by the Lol system (LolABCDE) and transported to the OM. Lpp and Pal are two OM lipoproteins that tether the OM to the PGN layer. Pal also binds to TolB, which also can interact with Lpp and OmpA, an OM β-barrel protein that can also associate with PGN (not shown).

Lipoprotein biosynthesis in Gram-negative bacteria is mediated by three IM-localized enzymes: Lgt, LspA, and Lnt (Fig. 1). All preprolipoproteins contain a signal peptide followed by a conserved four-amino-acid sequence, [LVI][ASTVI][GAS]C, also known as a lipobox (5). After secretion of preprolipoproteins through the IM via the Sec or Tat pathways, Lgt catalyzes the attachment of a diacylglyceryl moiety from phosphatidylglycerol to the thiol group of the conserved +1-position cysteine via a thioether bond (6). The second enzyme, prolipoprotein signal peptidase (LspA), is an aspartyl endopeptidase which cleaves off the signal peptide N-terminal to the conserved diacylglyceryl-modified +1 cysteine (7) and is the molecular target of the Gram-negative-specific natural-product antibiotics globomycin and myxovirescin (8–11). In Gram-negative and high-GC-content Gram-positive bacteria, a third enzyme, lipoprotein *N*-acyl transferase (Lnt), catalyzes the addition of a third acyl chain to the amino group of the *N*-terminal cysteine via an amide linkage. Mature triacylated lipoproteins destined for the OM are extracted from the IM by the LolCDE ATP-binding cassette (ABC) transporter and transported to the OM via the periplasmic chaperone protein LolA and the OM lipoprotein LolB (12, 13) (Fig. 1).

Two OM lipoproteins, Lpp (also known as murein lipoprotein or Braun’s lipoprotein) and Pal (peptidoglycan-associated lipoprotein), mediate tethering of the PGN layer to the OM in Escherichia coli. Lpp is a small ∼8-kDa lipoprotein that is the most abundant OM protein in E. coli (∼500,000 molecules per cell), and a third of all Lpp is covalently linked to PGN (2, 14). E. coli mutants deficient in Lpp exhibit increased sensitivity to ethylenediaminetetraacetic acid and SDS, leakage of periplasmic components, increased outer membrane vesicle (OMV) release, and increased sensitivity to complement-mediated lysis (15–17). Mislocalization and accumulation of PGN-linked Lpp in the IM upon inhibition of LspA (11, 18) and LolCDE (19, 20) is believed to lead to bacterial cell death (21–23). In addition to Lpp, Pal binds PGN and interacts with OmpA, Lpp, and the Tol complex and is crucial for maintaining OM integrity in E. coli (24–27). While LspA and LolCDE inhibitors were previously identified (19, 28), no inhibitors of the first committed step in bacterial lipoprotein biosynthesis have been described. Since many natural product antibiotics, including those that inhibit LspA, are cyclic (29, 30), we screened a macrocyclic peptide library to identify Lgt inhibitors. In this study, we identify and characterize G2824 as the first-described inhibitor of Lgt that inhibits growth of wild-type E. coli and Acinetobacter baumannii strains. In comparison to the downstream inhibition of LspA by globomycin, we demonstrate that either Lgt depletion or pharmacologic inhibition of Lgt by G2824 does not lead to significant accumulation of PGN-linked Lpp in the IM and, as such, is not sensitive to rescue of bacterial growth mediated by deletion of *lpp*.

## RESULTS

### Modest depletion of Lgt leads to loss of bacterial viability that is insensitive to *lpp* deletion.

**FIG 2:**
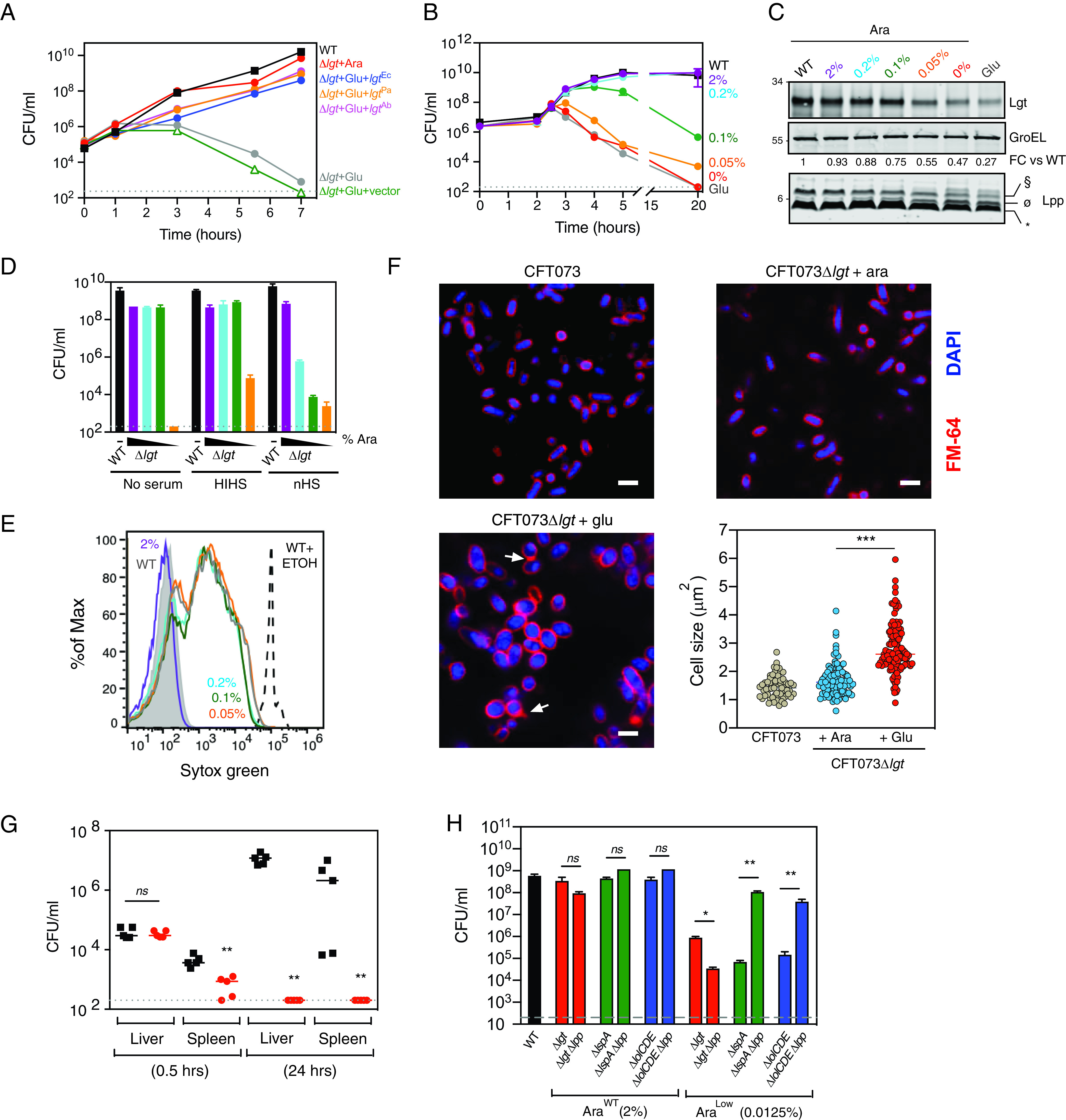
Lgt is essential for *in vitro* growth, membrane integrity, serum resistance and virulence. (A) CFT073 Δ*lgt* cells were grown in the presence of 4% arabinose (red) or 0.2% glucose (gray), and CFU were enumerated over 7 h posttreatment. CFT073 Δ*lgt* cultured in the presence of 0.2% glucose was complemented with empty pLMG18 plasmid (green) or pLMG18 plasmids expressing *lgt* from E. coli (blue), A. baumannii (magenta), or P. aeruginosa (orange). The dashed line represents the limit of detection (200 CFU/ml) of the experiment. (B and C) A modest reduction in Lgt levels results in a significant loss in viability over time with a concurrent accumulation of the unmodified pro-Lpp (ø; UPLP). CFT073 Δ*lgt* cells were treated with a range of arabinose concentrations, and CFU were enumerated over 20 h. CFU growth data are from experiments performed in duplicates. Western blot analysis for expression of Lgt and Lpp was performed using WT CFT073 and CFT073 Δ*lgt* total cell lysates harvested at 3 h post-arabinose treatment. To quantitate Lgt expression levels, Lgt levels were normalized to GroEL and quantitated as fold change relative to that of WT CFT073 (FC versus WT). Lpp forms are denoted as follows: *, triacylated free Lpp; §, PGN-linked diacylglyceryl pro-Lpp (DGPLP); ø, unmodified pro-Lpp (UPLP). Data are representative of two independent experiments. (D) Lgt depletion leads to increased serum sensitivity. WT CFT073 and CFT073 Δ*lgt* cells grown in the presence of a range of arabinose concentrations (2%, magenta; 0.2%, light blue; 0.1%, green, and 0.05%, orange) were incubated with 50% normal human serum (nHS), heat-inactivated human serum (HIHS), or medium (no serum) for 1 h, and CFU were enumerated. Data are representative of at least three independent experiments, each performed in duplicates. (E) Lgt depletion leads to increased OM permeability. WT CFT073 and CFT073 Δ*lgt* cells were incubated with the same range of arabinose concentrations as in panel D and incubated with the nucleic acid dye SYTOX green; flow cytometry was performed to determine the level of dye incorporation. While SYTOX green does not efficiently incorporate in bacterial cells with an intact OM (CFT073 Δ*lgt* treated with 2% arabinose, magenta), SYTOX green incorporation in bacterial cells increases after Lgt depletion. Intact CFT073 (WT; gray) or CFT073 treated with 70% ethanol (WT+ETOH; black), which permeabilizes the cells, were used as controls. Data are representative of two independent experiments. (F) Lgt depletion results in a globular cellular phenotype and membrane blebbing. WT CFT073 or CFT073 Δ*lgt* cells were grown in either arabinose or glucose for 4 h, fixed, and incubated with FM-64 dye (red) and DAPI (blue) to detect OM and nucleic acids, respectively. Cells were visualized by confocal microscopy. Arrows represent membrane blebs. Bars, 1 μm. Quantitation of cell size was performed using ImageJ software. (G) Lgt depletion leads to significant attenuation in virulence. Intravenous infection of neutropenic A/J mice with WT CFT073 (black) or CFT073 Δ*lgt* (red) cells. At 0.5 h and 24 h postinfection, bacterial burden in the liver and spleen was enumerated. Overall *P* value for the analysis of variance (ANOVA) is <0.0001. Pairwise comparisons were analyzed using unpaired Mann-Whitney test (**, *P* = 0.0079). The dashed line represents the limit of detection (200 CFU/ml) for this experiment. (H) Deletion of *lpp* does not rescue growth after Lgt depletion. E. coli MG1655 (WT, black) or inducible deletion strains for *lgt* (Δ*lgt*), *lspA* (Δ*lspA*) and *lolCDE* (Δ*lolCDE*) that either contained *lpp* or had *lpp* deleted were grown under conditions that allowed normal growth (Ara^WT^, 2% arabinose) or decreased growth (Ara^Low^, 0.0125% arabinose), and CFU were enumerated at 5 h posttreatment. Data are representative of two independent experiments, each performed in duplicates. ns, not significant; *, *P* < 0.05; **, *P* < 0.01.

Previous investigations into the role of Lgt in E. coli have focused on laboratory strains, specifically, those lacking the O-antigen of LPS. Here, we engineered the uropathogenic E. coli clinical isolate CFT073 so that the only copy of *lgt* was under the control of an arabinose-inducible promoter (CFT073 Δ*lgt*) and hence requires arabinose for Lgt expression. As expected, genetic depletion of Lgt was lethal *in vitro*, and growth was rescued after complementation with *lgt* from either E. coli, Pseudomonas aeruginosa PA14, or A. baumannii ATCC 17978 (which have 51.6% and 48.6% sequence identity to E. coli *lgt*, respectively) (Fig. 2A; see Fig. S1A in the supplemental material). *thyA*, the gene that encodes thymidylate synthase, is downstream of *lgt*, and its ribosome binding site overlaps the *lgt* stop codon. We confirmed that *thyA* expression, which is regulated by transcription from the *lgt* promoter and translational coupling (31), was unaffected after Lgt depletion (Fig. S1B). Overexpression of the E. coli genes encoding the downstream enzymes in lipoprotein biosynthesis (*lspA* and *lnt*) and transport (*lolCDE*) did not rescue growth of CFT073 Δ*lgt* in spite of detectable levels of LspA, Lnt, and LolCDE (Fig. S1C to G). We next determined how much depletion of Lgt was needed to cause cell death and lead to accumulation of unmodified pro-Lpp (UPLP), a major substrate of Lgt. Incubating cells with various arabinose concentrations led to a dose-dependent depletion of Lgt, UPLP accumulation, and decreased bacterial viability (Fig. 2B and C). While depletion of ∼25% of Lgt was sufficient for loss of cell viability (Fig. 2B and C), CFT073 Δ*lgt* cells expressing as high as ∼90% of normal levels of Lgt were significantly more sensitive to complement-mediated killing of the normally serum-resistant E. coli CFT073 (Fig. 2D) and showed increased incorporation of SYTOX green, a dye that normally does not penetrate an intact OM (Fig. 2E). Depletion of Lgt also resulted in an increase in cell size and an Lpp-dependent IM contraction due to osmotic stress (Fig. 2F and S2), as previously reported (32–34). Consistent with these results, partial depletion of Lgt led to increased sensitivity to antibiotics that are normally excluded by the impermeable Gram-negative OM in spite of normal growth *in vitro* (Table 1). Depletion of Lgt also resulted in significant attenuation in a mouse E. coli bacteremic infection model (Fig. 2G). Cumulatively, these data suggest that Lgt could be an attractive antibiotic target, since partial inhibition of Lgt may be sufficient to lead to significant attenuation of growth and cellular morphology.

**TABLE 1:** Antibiotic sensitivity of WT CFT073 versus CFT073 Δ*lgt* cells expressing wild-type (4% Ara) or low (0.25% Ara) levels of Lgt

Antibiotic	MIC (μM)^*a*^ for:		
	WT CFT073	CFT073 Δ*lgt*	
		Lgt^4% Ara^	Lgt^0.25% Ara^
Vancomycin	>100	>100	12.5
Rifamycin	6.3	6.3	0.8
Penicillin G	>50	>50	0.8
Oxacillin	>100	>100	12.5
Zeocin	12.5	12.5	0.8
Norfloxacin	0.4	0.6	0.2

a MIC values are averages from two independent experiments, each performed in duplicates.

Since bactericidal activity of LspA and LolCDE inhibitors is sensitive to deletion of the gene encoding the major OM lipoprotein, Lpp (18, 19), we wanted to determine if *lpp* deletion rescued bacterial cell growth after Lgt depletion. To compare growth to *lspA* and *lolCDE* inducible deletion strains, we constructed a *lgt* inducible deletion strain in E. coli MG1655 with and without *lpp* (MG1655 Δ*lgt* and MG1655 Δ*lgt* Δ*lpp*). Expectedly, *lpp* deletion rescued the growth of the *lspA* and *lolCDE* inducible deletion strains after depletion of LspA and LolCDE, respectively (Fig. 2H). In contrast, the *lpp* mutant was more sensitive to Lgt depletion, leading to a greater loss of CFU. Since the loss of *lpp* is a primary mechanism of resistance to inhibitors of LspA and LolCDE, identification of Lgt inhibitors would serve to confirm these genetic data and aid in identifying novel antibacterials.

### Identification and characterization of G2824 as a macrocyclic peptide inhibitor of Lgt.

**FIG 3:**
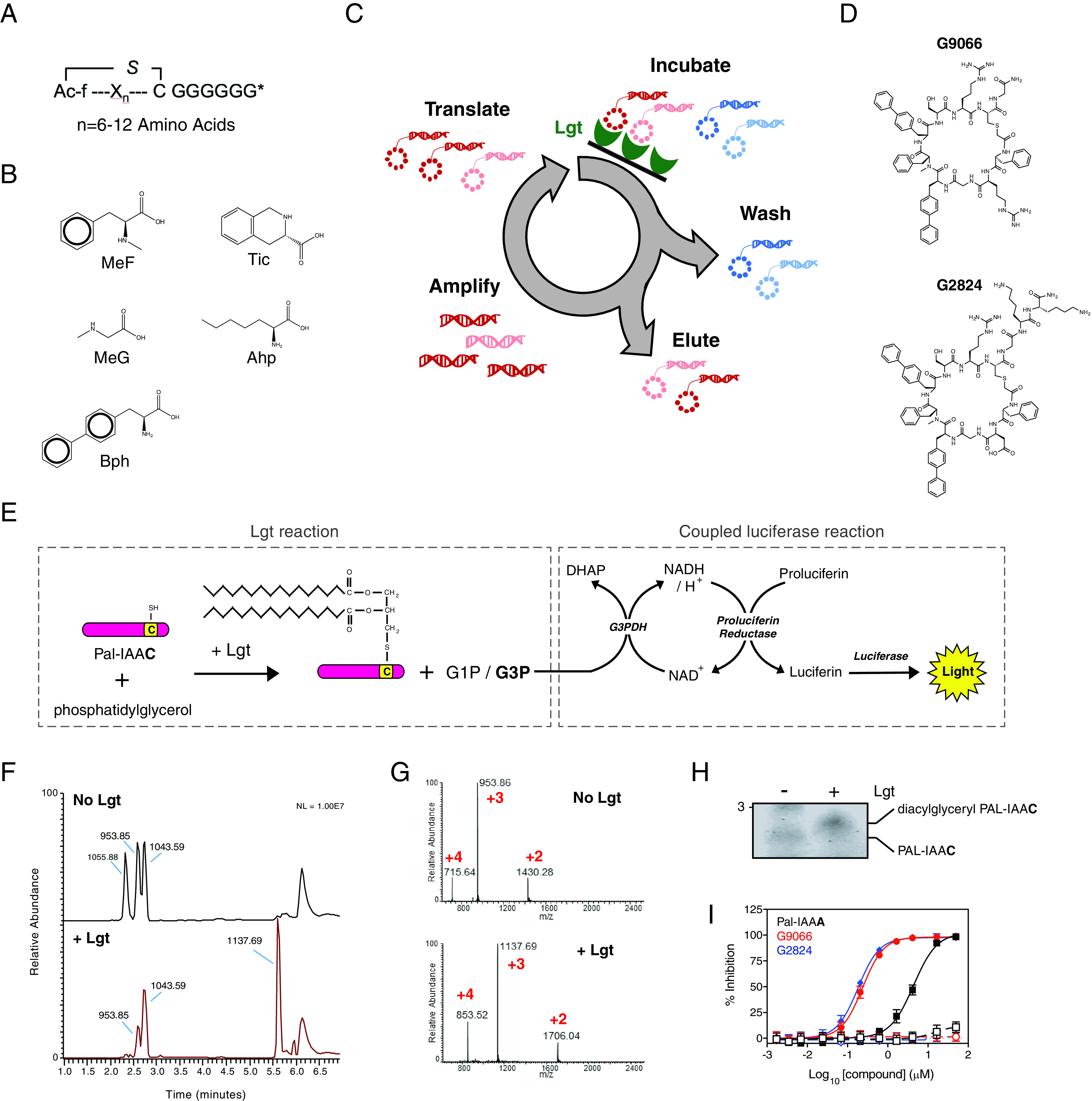
G9066 and G2824 macrocyclic peptides inhibit Lgt enzymatic activity. (A) Representation of macrocycle peptide libraries ranging in length from 8 to 14 amino acids. The variable region (X_n_) of the macrocycle libraries was encoded to allow the random incorporation of 11 natural amino acids and 5 nonnatural amino acids. (B) The 5 nonnatural amino acids used in the generation of the libraries were *N*-α-methyl-l-phenylalanine (MeF), *N*-α-methyl-l-glycine (MeG, sarcosine), (*S*)-2-aminoheptanoic acid (Ahp), 4-phenyl-l-phenylalanine (Bph), and (*S*)-1,2,3,4-tetrahydroisoquinoline-3-carboxylic acid (Tic). (C) Schematic representation of affinity-based selections using recombinant biotinylated Lgt immobilized on streptavidin magnetic beads. As discussed in Materials and Methods, Lgt-DDM was incubated with the macrocycle library and Lgt binders were eluted, amplified and translated to generate new libraries enriched for Lgt binders. Iterative rounds of affinity selection and washing were performed against recombinant Lgt and macrocycles that bound to Lgt were identified using next-generation sequencing. (D) Structure of the macrocyclic peptides G9066 and G2824 identified in this study. (E) Schematic of the *in vitro* Lgt biochemical Lgt reaction and coupled luciferase reaction. Lgt was incubated with phosphatidylglycerol and the Pal-IAAC peptide substrate for 60 min at RT, as described in Materials and Methods. As phosphatidylglycerol substrate used in our biochemical assay contains a racemic glycerol moiety at the end of phosphatidyl group, transfer of diacylglyceryl from phosphatidylglycerol to Pal-IAAC leads to the release of both G1P and G3P. In the coupling reaction, G3P is quantitatively converted to dihydroxyacetone phosphate (DHAP) with concomitant formation of an equivalent amount of NADH by the action of glycerol-3-phosphate dehydrogenase (G3PDH). Newly formed NADH, in turn, quantitatively reacts with proluciferin by the action of proluciferin reductase to generate equivalent amounts of luciferin, which ultimately results in proportional luminescence by luciferase. (F) Mass spectrometric confirmation of diacylglyceryl modification of the Pal-IAAC peptide by Lgt. Spectra depict the base peak chromatograms of the Pal-IAAC peptide ± Lgt. (G) MS^1^ spectra of the Pal-IAAC peptide in absence (2.59-min peak, top) or presence (5.61-min peak, bottom) of 100 nM Lgt. (H) Lgt reactions in the presence or absence of Lgt were analyzed by SDS-PAGE followed by staining with Coomassie blue. The slower migrating form, corresponding to the diacylglyceryl-modified Pal-IAAC peptide, was detected only in the presence of Lgt. (I) Dose-dependent inhibition of Lgt biochemical activity. Lgt reaction mixtures containing phosphatidylglycerol and Pal-IAAC were incubated in the presence or absence of G9066 (red), G2824 (blue), or a mutant Pal-IAAA peptide (black), which has the conserved cysteine mutated to alanine. Luminescence values were normalized to those of DMSO controls (0% inhibition) and no enzyme controls (100% inhibition). Negative-control reactions for each inhibitor were run in the absence of Lgt (open symbols). Data are representative of at least two independent experiments, each performed in triplicates.

Since many launched drugs are cyclic natural products or derivatives thereof (29), we screened a macrocyclic peptide library to identify specific and high-affinity binders of Lgt. We used a genetically reprogrammed *in vitro* translation system combined with mRNA affinity selection methods to generate large macrocycle peptide libraries with sizes ranging from 8 to 14 amino acids in length (35–37) (Fig. 3A). The variable sequence (6 to 12 amino acids) of the macrocycle libraries encoded the random incorporation of 11 natural amino acids (Ser, Tyr, Trp, Leu, Pro, His, Arg, Asn, Val, Asp, and Gly) and 5 nonnatural amino acids (Fig. 3B). The library screening is schematically depicted in Fig. 3C. Biotinylated Lgt was solubilized in 0.02% *n*-dodecyl β-d-maltoside (DDM), immobilized on streptavidin magnetic beads, and incubated with the macrocyclic library. Iterative rounds of affinity selection were performed to identify Lgt-binding macrocycles. After five rounds of enrichment, two additional rounds of off-rate selections were performed by increasing the stringency of the washes before high-affinity binders were eluted. Hit macrocycles were identified using next-generation sequencing (NGS) on the last four rounds of selection followed by a frequency analysis calculation. Two macrocycles, 692 and 693 (see Fig. S3A), were significantly enriched in the final round of selection, accounting for 19.4% and 10.1%, respectively, of peptide sequences as measured by NGS. Macrocycles 692 and 693 each contain 7 amino acids, are related to one another with a charge swap at position 2, and have molecular weights (MWs) of 1428.66 and 1259.55, respectively. The calculated values for LogP (cLogP), which is the logarithm of the compound’s partition coefficient between *n*-octanol and water and a measure of a molecule’s hydrophilicity, were 1.8 and 1.7 for 692 and 693, respectively. Macrocycle 692 was synthesized with a Gly off the C terminus and renamed G9066 (Fig. 3D). Macrocycle 693 was synthesized with a Gly-Lys-Lys tail off the C terminus to aid in solubility and was renamed G2824 (Fig. 3D).

We then tested the ability of G9066 and G2824 to inhibit E. coli Lgt enzymatic activity *in vitro* by measuring the release of glycerol phosphate, which is a by-product released from the Lgt-catalyzed transfer of diacylglyceryl from phosphatidylglycerol to a peptide substrate via formation of a thioether bond (Fig. 3E). The peptide substrate was derived from the Pal lipoprotein (Pal-IAAC, where C is the conserved cysteine that is modified by Lgt). While glycerol-1-phosphate (G1P) is the expected by-product of the Lgt enzymatic activity (6), the phosphatidylglycerol substrate used in our biochemical assay contains a racemic glycerol moiety at the end of the phosphatidyl group; hence, both G1P and glycerol-3-phosphate (G3P) are released from phosphatidylglycerol as Lgt catalyzes the reaction (Fig. 3E). The detection of G3P is based on a coupled luciferase reaction (described in Materials and Methods and Fig. 3E). Using mass spectrometry, we determined that the addition of 552 Da to the Pal-IAAC peptide, which corresponds to the diacylglyceryl moiety, is Lgt dependent (Fig. 3F and G and S3B and C). Furthermore, SDS-PAGE analysis of the Lgt reaction confirmed that detection of a slower-migrating Pal-IAAC species, corresponding to diacylglyceryl Pal-IAAC, is Lgt dependent (Fig. 3H). G9066 and G2824 potently inhibit Lgt biochemical activity (50% inhibitory concentrations [IC_50_s] of 0.24 μM and 0.18 μM, respectively) (Fig. 3I). In comparison, a mutant Pal peptide substrate with the conserved cysteine mutated to alanine (Pal-IAAA), which cannot be modified and acts as a Lgt-binding nonreactive, substrate-based competitive inhibitor (Fig. S3D), inhibits Lgt with an IC_50_ of 4.4 μM (Fig. 3F). Generation of G3P was dependent on Lgt, as reactions performed in the absence of Lgt (Fig. 3F) or using heat-inactivated Lgt (Fig. S3E) did not yield a luciferase signal. Furthermore, G9066 and G2824 did not inhibit steps involved in the coupling reaction (Fig. S3F) or the background signal (Fig. S3G), demonstrating that the inhibitory effects observed with G9066 and G2824 were specifically due to inhibition of the Lgt-catalyzed enzymatic reaction.

G9066 and G2824 were then tested against bacterial cells in MIC growth assays. While G9066 and G2824 only modestly inhibited growth of WT E. coli MG1655 and CFT073 (MIC, ∼100 μM), both molecules showed greater growth inhibition of the *lpp*-deleted strains (MG1655 Δ*lpp* and CFT073 Δ*lpp*) (Table 2). In contrast, the *lpp*-deleted MG1655 and CFT073 strains were resistant to the natural product inhibitor of LspA, globomycin, as expected. Both G9066 and G2824 also inhibited growth of wild-type (WT) A. baumannii 19606, with a MIC of 37.5 μM. Using bacterial cells treated with EDTA or expressing the *imp4213* allele of *lptD*, which leads to permeabilization of the OM (38), we demonstrate that G9066 and G2824 inhibit growth of OM-permeabilized E. coli, P. aeruginosa PA14, and A. baumannii 19606 (Table 2). Consistent with data from the WT strains, *lpp* deletion in the context of the *imp4213* or EDTA treatment led to a modest increase in G9066 and G2824 antibacterial activity, unlike that seen with globomycin (Table 2). G2824 showed minimal nonspecific activity against eukaryotic cells and the Gram-positive Staphylococcus aureus strain USA300, consistent with data demonstrating *lgt* is dispensable for Gram-positive bacterial growth *in vitro* (39). In contrast, G9066 inhibited growth of USA300 to a greater extent, suggesting G9066 may have additional targets or nonspecific cellular effects. Given G9066 and G2824 are structurally very similar, we decided to focus on G2824 for the remainder of this study.

**TABLE 2:** Growth inhibition of a panel of bacterial strains and eukaryotic cells by G9066, G2824, globomycin, and vancomycin

Bacterium or effect	Strain and treatment	MIC or EC_50_ (μM)^*a*^			
		G9066	G2824	Globomycin	Vancomycin
E. coli	MG1655	>100	93.8	25	>100
	MG1655 Δ*lpp*	31.3	62.5	50	>100
	MG1655 + EDTA	3.1	3.1	0.8	3.1
	MG1655 Δ*lpp* + EDTA	3.1	3.1	12.5	0.8
	CFT073	100	92	29.2	>100
	CFT073 Δ*lpp*	31.3	62.5	>100	>100
	CFT073 + EDTA	3.1	3.1	1.1	0.8
	CFT073 Δ*lpp* + EDTA	1.6	2.3	4.7	0.8
	CFT073 *imp4213*	5.7	8.4	0.5	0.6
	CFT073 *imp4213* Δ*lpp*	6.8	8.9	11.5	0.7
A. baumannii	19606	37.5	37.5	25	>100
	19606 + EDTA	3.1	6.3	0.6	0.2
P. aeruginosa	PA14	>100	>100	>100	>100
	PA14 *imp4213*	6.3	6.3	50	12.5
	PA14 + EDTA	6.3	6.3	50	6.3
S. aureus	USA300	6.3	>100	>100	0.4
Mammalian cytotoxicity^*b*^	HepG2	>100	>100	>100	>100
	Hela	>100	>100	>100	>100
	293T	>100	>100	>100	>100

a All E. coli MIC values represent averages from at least four independent experiments, each performed in duplicates. For other bacterial strains, MIC values represent averages from two independent experiments each performed in duplicates.

b Mammalian cytotoxicity (EC_50_) values are representative of three independent replicates.

### G2824 targets Lgt function in E. coli, leading to growth inhibition.

**FIG 4:**
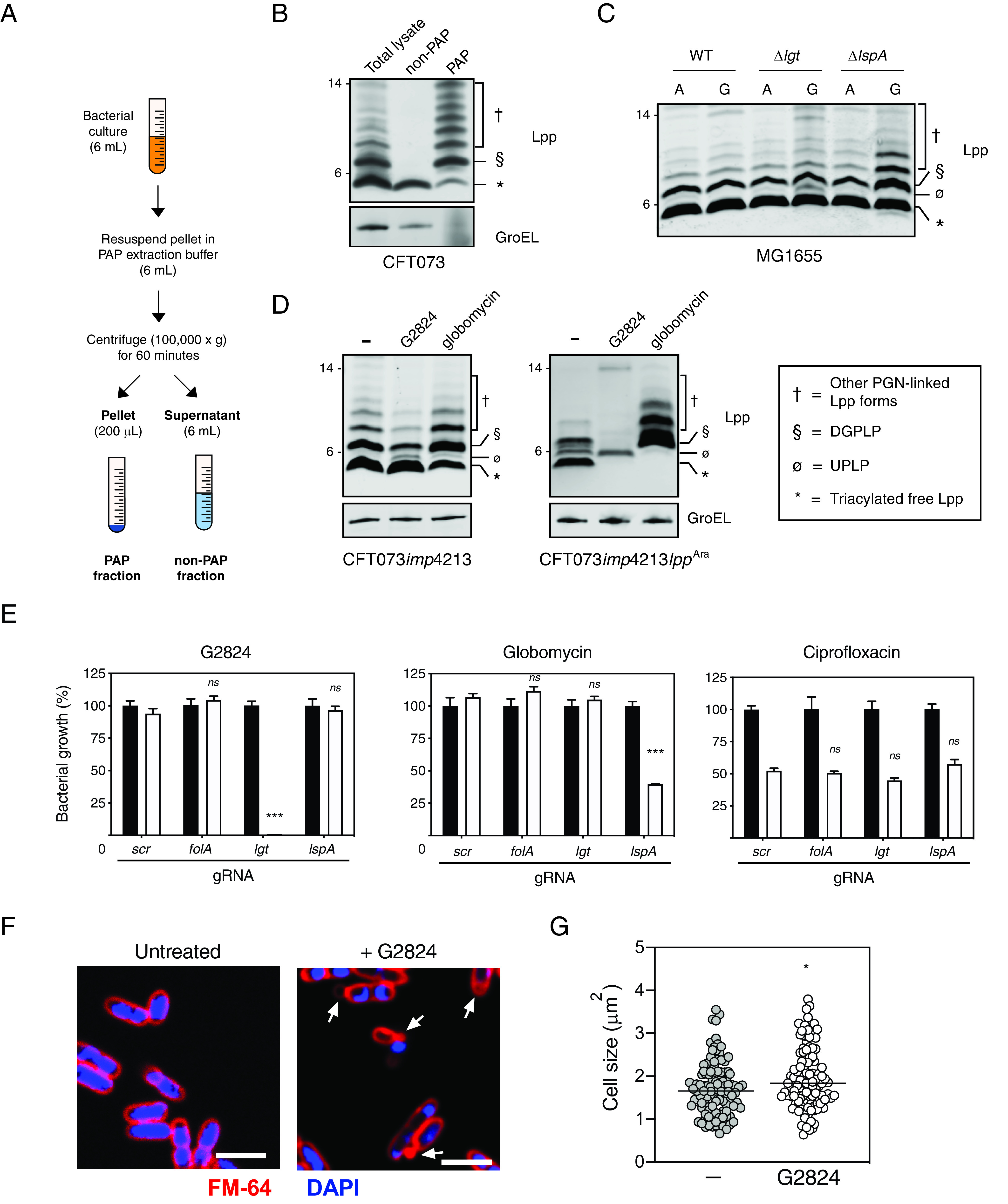
G2824 inhibits Lgt enzymatic activity in bacterial cells. (A) Schematic representing the isolation of PAP and non-PAP fractions. Bacterial cultures were resuspended in 6 ml of PAP extraction buffer and centrifuged at 100,000 × *g* for 60 min. Six milliliters of supernatants was collected, and pellets were resuspended in 200 μl of PAP extraction buffer. (B) SDS fractionation of WT CFT073 cells to distinguish PGN-associated versus non-PGN-associated forms of Lpp. CFT073 *imp4213* cells were treated with SDS to enrich for PAP and non-PAP fractions as discussed in Materials and Methods, and Western blot analysis was performed to detect levels of Lpp. GroEL was used as a control for enrichment of the PAP fraction. While triacylated free Lpp (*) was enriched in the SDS-soluble non-PAP fraction, higher-molecular-weight Lpp species (§ and †) were enriched in the SDS-insoluble PAP fraction (§, DGPLP; †, other PGN-linked Lpp forms). Molecular weight markers (in kilodaltons) are denoted on the left of the blots. (C) Detection of Lpp intermediates in WT MG1655, MG1655 Δ*lgt* and MG1655 Δ*lspA* inducible deletion strains by Western blotting. WT or inducible deletion strains were treated with 2% arabinose (A) or 0.2% glucose (G), and total cell lysates were harvested at 3 h posttreatment (Ø, unmodified pro-Lpp [UPLP]). PGN-linked DGPLP (§) and other higher-molecular-weight Lpp species (†) accumulated after LspA depletion. (D) G2824 treatment leads to accumulation of UPLP. CFT073 *imp4213* cells were treated with 0.5× MICs of G2824 or globomycin for 30 min, and Western blot analysis was performed to measure levels of Lpp forms, which are denoted as described above and in the figure. (E) CRISPRi knockdown of *lgt* gene expression sensitized cells to G2824 but not globomycin. E. coli BW25113 cells expressing dCas9 and gRNAs specific to *lgt* or *lspA* were untreated (black bars) or treated (white bars) with 2 μM G2824, 0.05 μM globomycin or 0.016 μM ciprofloxacin. A scrambled (scr) gRNA and gRNA specific to *folA* (dihydrofolate reductase) were used as negative controls. Bacterial growth was measured by OD_600_, and values were normalized to the untreated sample for each gRNA, which was set at 100%. *P* values were determined comparing to scr gRNA-expressing cells treated with the specific inhibitor (***, *P* < 0.001). Data are representative of at least two independent experiments, each performed in triplicates. (F) G2824 treatment leads to cell morphology changes and membrane blebs. CFT073 *imp4213* cells were left untreated or treated with G2824 for 30 min, fixed, and incubated with FM-64 dye (red) and DAPI solution (blue) to stain membranes and nucleic acid, respectively, and visualized by confocal microscopy. Arrows represent membrane blebs. Bars, 3 μm. (G) Quantitation of cell size after treatment with G2824. A total of 104 ± 4 cells per treatment were quantitated using ImageJ. *, *P* = 0.04; ***, *P* = 0.002.

While G2824 inhibited both Lgt enzymatic function and bacterial growth, it was unclear whether inhibition of bacterial cell growth was mediated by specific inhibition of Lgt function. While G2824 solubility issues prevented performing agar plate-based resistance selections, multistep resistance selections did not identify on-target resistant mutants to G2824 in CFT073 *imp4213*. Resistant mutations in *lptD* (D322V or N274I) were identified, and since N274I was previously demonstrated to be a suppressor of the *imp4213* phenotype (38), we believe the mutations identified were nonspecific suppressors leading to increased OM integrity. Therefore, multiple experimental approaches were undertaken to determine if inhibition of bacterial growth was indeed Lgt dependent. As the accumulation of Lpp intermediates detected by Western blotting has been successfully used to confirm inhibition or deletion of enzymes in lipoprotein biosynthesis or transport (20, 21), we asked if Lgt treatment led to the accumulation of UPLP, the pro-Lpp substrate of Lgt. We initially sought to verify the various Lpp forms by leveraging a previously validated protocol using SDS fractionation (17, 40, 41), which separates the SDS-insoluble PGN-associated proteins (PAP) and SDS-soluble non-PGN-associated proteins (non-PAP) (Fig. 4A). Lysozyme was added to allow for the identification of PGN-linked Lpp forms, as previously demonstrated (42). As expected, the fastest migrating form representing the triacylated mature form of Lpp (*) was enriched in the non-PAP fraction, and the PGN-linked Lpp forms (†) were enriched in the PAP fraction (Fig. 4B). We also detected a form corresponding the diacylglyceryl modified pro-Lpp (DGPLP; §) enriched in the PAP fraction, which was previously reported to be PGN linked (42). We then asked if we could detect UPLP in total cell lysates after Lgt depletion in the *lgt* inducible deletion strain. We confirmed that specific depletion of Lgt led to the accumulation of the UPLP (Fig. 4C, ø), consistent with previous results (43). Depletion of LspA but not Lgt led to the accumulation of DGPLP (§) and other PGN-linked Lpp forms (†) (Fig. 4C). These results now enabled us to determine whether accumulation of UPLP was also detected after treatment with G2824.

Since G2824 has moderate activity against WT bacterial strains, we performed the mechanistic studies in CFT073 *imp4213*, as Lgt depletion in CFT073 *imp4213* (CFT073 *imp4213* Δ*lgt*) leads to a similar extent of Lgt depletion, loss in viable CFU, and accumulation of UPLP as seen in CFT073 Δ*lgt* (compare Fig. S4A and B and Fig. 2B and C), confirming that CFT073 *imp4213* cells are not more sensitive to Lgt inhibition. G2824 treatment of CFT073 *imp4213* led to an accumulation of UPLP (Fig. 4D, ø), consistent with what was observed with the *lgt* inducible deletion strains in both the WT background and *imp4213* backgrounds. As expected, treatment with globomycin led to an accumulation of DGPLP and other PGN-linked Lpp forms. Levels of triacylated free Lpp were not significantly decreased after either depletion or inhibition of Lgt or LspA (Fig. 4C and D), likely due to the presence of high levels of mature triacylated Lpp expressed in the OM. We engineered CFT073 *imp4213* to express Lpp in an arabinose-inducible manner (CFT073 *imp4213* *lpp^Ara^*) to demonstrate that G2824, like globomycin, prevented the formation of mature triacylated Lpp. Lpp expression was induced prior to treatment with G2824 or globomycin to minimize the level of preexisting triacylated Lpp. G2824 treatment of CFT073 *imp4213* *lpp^Ara^* resulted in accumulation of UPLP as seen in CFT073 *imp4213* and, like globomycin, led to decreased mature Lpp (Fig. 4D, right, *). These data demonstrate that G2824 identified in this study leads to the accumulation of Lgt substrate, UPLP, and inhibits the generation of mature triacylated Lpp.

Lgt-specific G2824 activity in bacterial cells was further confirmed using two additional methods. First, we asked whether cells expressing reduced levels of Lgt would be specifically sensitized to G2824 compared to the other inhibitors. To test this hypothesis, we utilized CRISPR interference (CRISPRi) technology to decrease gene expression of the enzymes involved in lipoprotein biosynthesis and transport. BW25113 cells containing plasmids expressing dCas9 and guide RNAs (gRNAs) specific to *lgt* and *lspA* were treated with G2824, globomycin, and a control antibiotic, ciprofloxacin, and bacterial growth was measured. gRNA-mediated downregulation of target gene expression (Fig. S4C and D) was consistent with published reports for CRISPRi in bacterial cells (44). Decreased *lgt* expression specifically sensitized cells to G2824 but not globomycin compared to that of the negative-control gRNAs targeting a scrambled sequence (scr) or the *folA* gene (Fig. 4E). As expected, decreased expression of *lspA* specifically led to enhanced growth inhibition by globomycin and not G2824 (Fig. 4E). In contrast, decreased *lgt* or *lspA* expression did not lead to increased sensitivity to ciprofloxacin compared to scr or *folA* gRNAs. Although not specific to targeting lipoprotein biosynthesis, G2824 treatment also led to the expected increase in cell size, as seen with inhibitors of LspA and LolCDE, and OM blebbing (Fig. 4F and G), as seen with the inducible deletion strain (Fig. 4F) and previously demonstrated in a Pal-deficient E. coli strain (45). Cumulatively, our data demonstrate that the novel Lgt-binding macrocycle G2824 interferes with Lgt activity, leading to inhibition of E. coli growth.

### Loss of peptidoglycan-linked Lpp enhances the rate of G2824 antibacterial activity.

**FIG 5:**
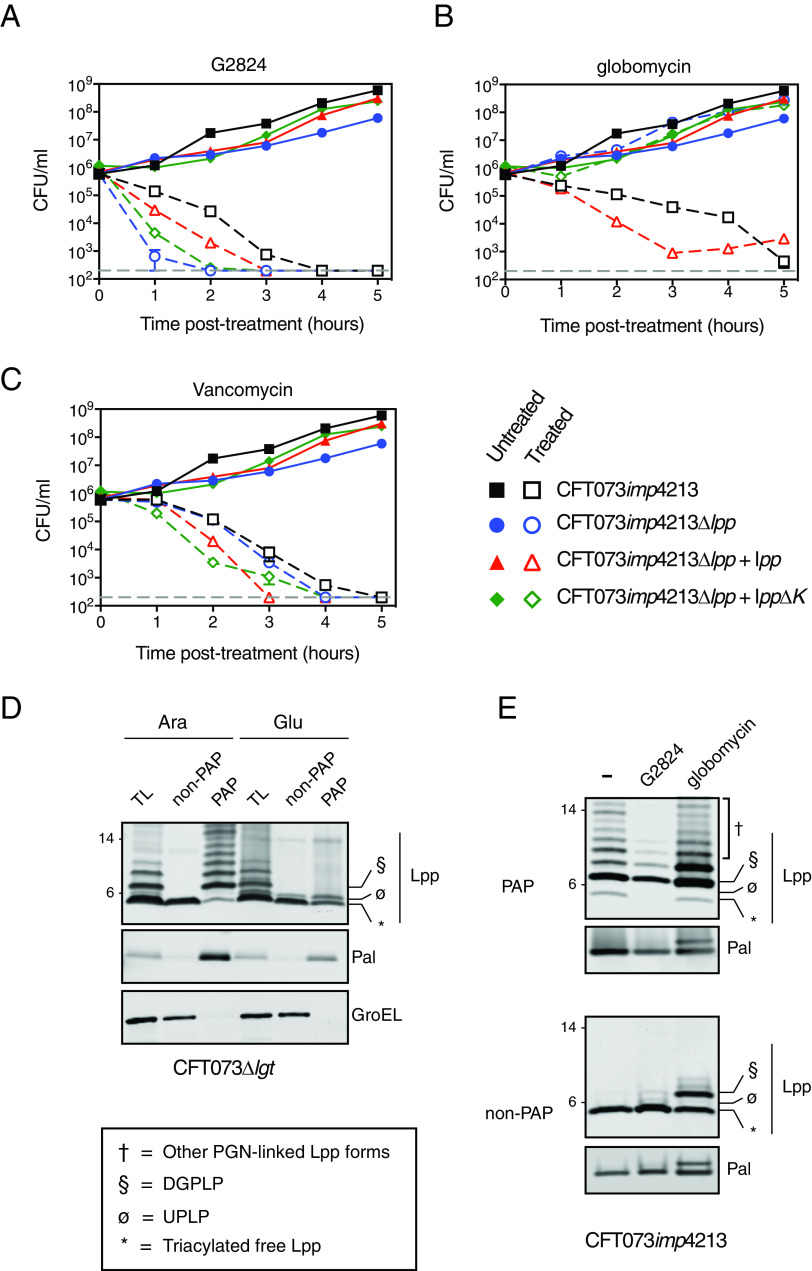
*lpp* deletion does not rescue growth after G2824 treatment. CFT073 *imp4213* (black), CFT073 *imp4213* Δ*lpp* (blue), or CFT073 *imp4213* Δ*lpp* complemented with pBAD24 plasmids encoding WT *lpp* (red) or *lpp*ΔK (green) were untreated or treated with 12.5 μM G2824 (A), 3.2 μM globomycin (B), or 1.6 μM vancomycin (C). CFU were enumerated at various times posttreatment. Data are representative of two independent experiments, each performed in triplicates. (D) Lgt depletion leads to loss of PGN-linked Lpp and Pal. CFT073 Δ*lgt* inducible deletion cells were grown in arabinose (Ara) or glucose (Glu), and Lpp and Pal expression was determined in total cell lysates (TL), SDS-insoluble (PAP), and SDS-soluble (non-PAP) fractions 5 h postincubation. GroEL was used as a control for fractionation. (E) G2824 treatment leads to decreased PGN-associated Lpp and Pal. CFT073 *imp4213* cells were untreated or treated with G2824 or globomycin for 60 min at 0.5× MIC, and levels of Lpp and Pal were measured in PAP and non-PAP fractions. These data are representative of two independent experiments. Lpp forms are denoted as described in the figure: *, triacylated free Lpp; §, DGPLP; ø, UPLP; †, other PGN-linked Lpp forms.

Our data with the inducible deletion strains (Fig. 2H) as well as MIC data (Table 2) suggested that *lpp*-deleted cells are more sensitive to Lgt depletion or Lgt inhibition, distinguishing it from the mechanism of cell death involved in the later steps of lipoprotein biosynthesis and transport. To compare the rate of bactericidal killing of G2824 with that of globomycin, we treated CFT073 *imp4213* and CFT073 *imp4213* Δ*lpp* cells with G2824 and globomycin at 2× MIC of the respective inhibitors against CFT073 *imp4213* and enumerated viable CFU counts. Consistent with our previous data, CFT073 *imp4213* Δ*lpp* cells were not protected from G2824, and, in fact, G2824 led to a more rapid loss of CFT073 *imp4213* Δ*lpp* viability than that of CFT073 *imp4213* (Fig. 5A). As expected, inhibition of bacterial growth by globomycin was lost in the absence of *lpp* (Fig. 5B). In contrast, vancomycin showed equivalent killing of CFT073 *imp4213* and CFT073 *imp4213* Δ*lpp* at 5 h posttreatment (Fig. 5C), confirming the vancomycin MIC data (Table 2) that the lack of *lpp* does not lead to nonspecific increases in OM permeability. These data confirm that *lpp* deletion is not a mechanism of resistance to G2824 and that loss of *lpp* leads to increased bactericidal activity of G2824.

To determine if loss of PGN linkage of Lpp leads to increased G2824 bactericidal activity, we treated CFT073 *imp4213* Δ*lpp* cells complemented with either WT *lpp* or a mutant form that is unable to covalently link to PGN (*lpp*ΔK). Using the previously described SDS fractionation protocol, we confirmed that while WT Lpp localized to both PAP and non-PAP fractions, the LppΔK mutant was primarily detected in the non-PAP fraction (see Fig. S5A). As we noted earlier, PGN-linked DGPLP (§) and other PGN-linked Lpp forms (†) were primarily detectable in the PAP fraction (Fig. S5A). Bacterial viability of cells not expressing *lpp* or only expressing the mutant *lpp*ΔK was lost at a higher rate than cells expressing WT *lpp* after treatment with G2824 (Fig. 5A). In comparison, cells expressing *lpp*ΔK were completely resistant to globomycin, similar to the *lpp*-deleted cells (Fig. 5B). These data suggest that PGN-linked Lpp is important for OM integrity after treatment with an Lgt inhibitor.

### Lgt depletion or inhibition leads to decreased PGN-linked Lpp.

Unlike with the LspA inhibitors globomycin and myxovirescin, *lpp* deletion does not rescue growth after treatment with G2824, suggesting that the PGN-linkage state and/or localization of Lpp must differ after G2824 treatment. Using SDS fractionation of CFT073 Δ*lgt* inducible deletion cells, we found that Lgt depletion led to significantly lower levels of PGN-linked Lpp, including DGPLP, in the PAP fraction (Fig. 5D). Lgt depletion also led to decreased PGN-associated Pal, likely due to decreased overall levels of triacylated Pal. We next tested if G2824 treatment also led to a similar loss of PGN-associated Lpp. As seen with the *lgt* inducible deletion strain, treatment with G2824 led to decreased levels of PGN-linked Lpp and Pal (Fig. 5E). Globomycin treatment led to an expected accumulation of PGN-linked DGPLP (§) and other PGN-linked Lpp forms (†). These data suggest that after Lgt inhibition, the accumulated Lgt substrate, UPLP, either is not sufficiently PGN-linked or does not accumulate in the IM to levels needed to cause cell lysis.

### Inhibition of Lgt does not lead to significant accumulation of PGN-linked Lpp in the IM.

**FIG 6:**
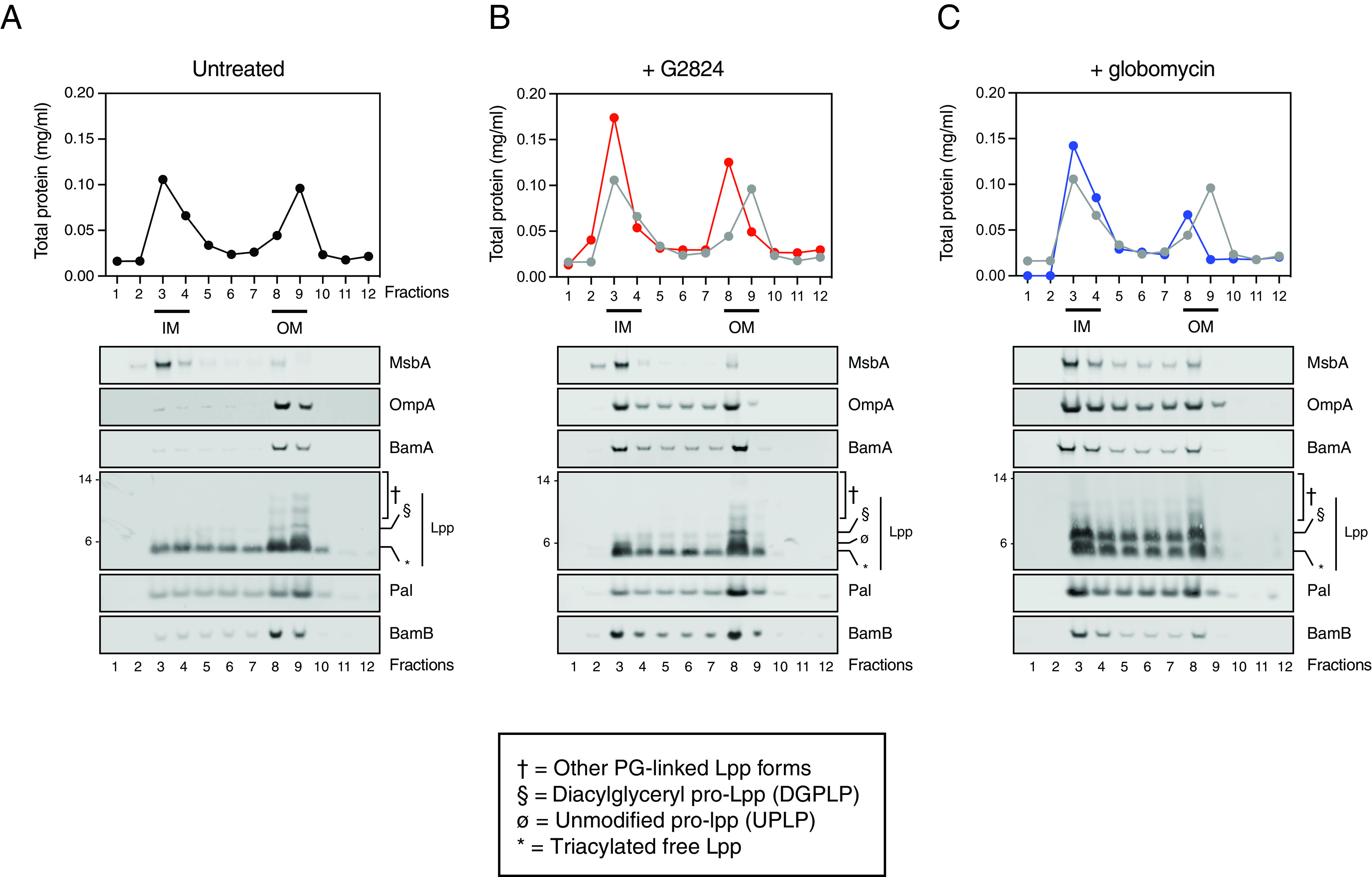
Inhibition of Lgt leads to depletion of essential OM lipoproteins and OMPs and minimal IM accumulation of PGN-linked DGPLP. MG1655 *imp4213* cells were left untreated (A) or treated with G2824 (B) or globomycin (C) for 60 min at 1× MIC and subjected to isopycnic sucrose gradient ultracentrifugation as described in Materials and Methods. Total protein in each fraction was quantitated. Total protein from G2824-treated (red) and globomycin-treated (blue) samples were compared to that in untreated cells (black in panel A; gray in panels B and C). IM and OM fractions were assigned based on the total protein peaks and expression of MsbA and OmpA. These data are representative of at least two independent experiments. *, triacylated free Lpp; §, DGPLP; ø, UPLP; †, other PGN-linked Lpp forms.

To determine if Lpp accumulated in the IM after G2824 treatment, we utilized sucrose gradient centrifugation to separate the IM and OM and measured levels of OM lipoproteins (Lpp, Pal, and BamB) and outer membrane proteins (OMPs) (BamA and OmpA) by Western blotting analyses. Separation of IM and OM from MG1655 *imp4213* was more efficient than that seen with CFT073 *imp4213* (Fig. 6A and S5B), as determined by probing sucrose gradient centrifugation fractions for MsbA, OmpA, and Lpp levels by Western blotting, and was therefore used to test the various inhibitors. In comparison to untreated cells, treatment with globomycin or G2824 led to increases in total protein levels in the IM fraction and a decrease in OM density (Fig. 6). This was further validated by demonstrating IM accumulation of OM lipoproteins (Pal and BamB) and OMPs (BamA and OmpA) after treatment with G2824 or globomycin (Fig. 6B and C). While globomycin treatment also led to a significant IM accumulation of PGN-linked Lpp, including DGPLP, no such accumulation of PGN-linked Lpp was detected after G2824 treatment. We did observe IM accumulation of UPLP after G2824 treatment (Fig. 6B). Since the DGPLP form of Lpp is PGN linked, our data suggest that the lack of significant IM accumulation of PGN-linked Lpp forms, including DGPLP, after G2824 treatment could explain why *lpp* deletion does not mediate resistance to Lgt inhibitors.

## DISCUSSION

Lipoprotein biosynthesis is a critical pathway involved in the biogenesis and maintenance of the Gram-negative bacterial OM, and disruption of any step in this pathway leads to loss of cell viability. Lpp maintains the integrity of the Gram-negative bacterial cell surface by covalent interaction between the C-terminal lysine and the *meso*-diaminopimelic acid residue of the PGN layer (15, 46–49). Published data suggest that *lpp* deletion leads to rescue of growth after inhibition of LspA and LolCDE (11, 18–20, 23) as well as rescue of the temperature-sensitive Salmonella enterica serovar Typhimurium *lgt* and *lnt* mutants (50, 51). While E. coli *lnt* is essential in the absence of *lpp* (22), Lpp overexpression in the E. coli *lnt* mutant leads to bacterial cell growth arrest (21). Following up on data from Pailler et al., who demonstrated that *lgt* is essential in BW25113, a derivative of E. coli K-12 strain BD792, and that Lgt depletion leads to increased DNA leakage from the cell pole (43), we demonstrate that Lgt depletion in the clinical E. coli strain CFT073 leads to significant perturbations to the bacterial cell envelope, leading to increased sensitivity to antibiotics, increased serum killing, and attenuated virulence *in vivo*.

Based on these published reports, we had expected that deletion of *lpp* would also lead to rescue of growth after Lgt depletion or pharmacologic inhibition of the enzyme. However, our data demonstrate that pharmacologic inhibition of Lgt by G2824 or Lgt depletion leads to cell death that is not rescued by *lpp* deletion in two E. coli strain backgrounds, CFT073 and MG1655. Furthermore, neither genetic depletion nor enzymatic inhibition of Lgt leads to significant accumulation of PGN-linked Lpp forms. While a previous report demonstrated that UPLP and DGPLP are both linked to a single muropeptide unit (42), our data suggest that the level of complete PGN linkage is likely less efficient in the absence of diacylglyceryl modification of pro-Lpp. While our findings suggest the lack of diacylglyceryl modification by Lgt may generate a less optimal substrate for the l,d-transpeptidases that covalently link Lpp to PGN, they do not rule out the possibility that PGN linkage occurs at or after modification by Lgt. Our data are in contrast to those generated by Zhang et al. (49), who immunoprecipitated [^35^S]methionine-labeled Lpp from JA221 E. coli cells to demonstrate that lipid modification of Lpp is not essential for linkage to PGN. While the explanation for this is unclear, this could be due, in part, to differences in E. coli strains used in both studies, as the same mutation generated in JA221 and MG1655, both K-12-derived E. coli strains, can lead to strikingly different sensitivities (∼4,000-fold) to bacterial cell stress (52). Both G2824 and globomycin treatment lead to mislocalization of the β-barrel protein, OmpA (Fig. 6B and C), which is likely due to decreased OM localization of BamB and other Bam lipoproteins that are crucial for optimal OM localization of BamA in E. coli. Since most of the studies with G2824 were performed in cells expressing the *lptD imp4213* mutant allele, identifying more-potent Lgt inhibitors will be needed to determine the effects of Lgt inhibition in WT bacterial cells. However, given that Lgt depletions in CFT073 and CFT073 *imp4213* show similar kinetics of cell viability loss, Lgt depletion, and accumulation of pro-Lpp (Fig. 2; see Fig. S4 in the supplemental material), we believe our results in the *imp4213* background should translate to WT E. coli. It is conceivable that Lpp normally protects cells against inhibitors of lipoprotein biosynthesis and transport by maintaining optimal OM structure and integrity but that this is masked in the case of LspA and LolCDE inhibitors, as they lead to the accumulation of PGN-linked Lpp, which itself induces cell death.

Using a combination of biochemical and genetic strategies, we believe the Lgt inhibitor G2824 identified in this study inhibits bacterial cell growth through inhibition of the diacylglyceryl transferase activity of Lgt. First, G2824 was identified using a specific Lgt binding assay and confirmed to inhibit Lgt enzymatic function *in vitro*. Second, the results generated from pharmacologic inhibition of Lgt by G2824 in both CFT073 and MG1655 E. coli strains are highly consistent with the genetic depletion of Lgt, strongly arguing against off-target effects as a main cause of G2824 bactericidal activity. Third, our sucrose gradient centrifugation data clearly demonstrate that G2824, like globomycin, leads to IM accumulation of OM lipoproteins (Pal and BamB) and OMPs that are dependent on lipoproteins for their localization (BamA). While the sucrose gradients clearly separate IM and OM in untreated cells, some overlap of the IM fractions is detected for G2824 and globomycin-treated cells. This is not unexpected and is detected in most published reports when using sucrose gradient centrifugation to examine membrane localization of Lpp in WT E. coli membranes (22, 23, 53), suggesting this is unlikely due to the use of *lptD imp4213*-expressing cells. Interestingly, separation of IM and OM in globomycin-treated cells was more challenging, likely explained by the significant accumulation of membranes as detected by electron microscopy after inhibition of LspA (54). While we were unable to raise on-target resistant mutants to G2824, we cannot fully rule out that G2824 does not have additional targets in bacterial cells. However, one could speculate that if G2824 binds to the conserved phosphatidylglycerol binding site in Lgt, mutations disrupting G2824 binding might be incompatible with bacterial viability. In fact, no on-target resistance mutations have ever been identified against the LspA inhibitor globomycin (55) or its improved analog, G0790 (54), which is believed to act as a substrate mimic and bind the highly conserved LspA active site (56). As recent publications have revealed significant insights into the potential mechanisms of diacylglyceryl modification by Lgt (57, 58), further studies aimed at determining if G2824 competitively inhibits binding of the phosphatidylglycerol or prolipoprotein substrates would be needed to better understand the mechanism by which G2824 interferes with this critical OM biogenesis pathway. Although we do not detect MIC shifts with G2824 in cells overexpressing *lgt* (data not shown), drug resistance in E. coli after target overexpression is known to vary (increase, remain unchanged, or decrease) depending on the balance between bacterial fitness costs and inhibition of enzymatic activity (59). One could speculate that even a modest inhibition of Lgt could lead to significant defects in OM integrity and cellular fitness, which may counteract any resistance arising from *lgt* overexpression.

In summary, our study is the first to systematically differentiate the role of Lpp in targeting multiple steps of bacterial lipoprotein biosynthesis. The lack of significant PGN association of Lpp, resulting from Lgt depletion or pharmacologic inhibition of the enzyme, eliminates a major mechanism of resistance mediated by deletion of *lpp* and, as such, could inform future antibacterial strategies to inhibit this essential OM biogenesis pathway.

## MATERIALS AND METHODS

### Ethics statement.

All mice used in this study were housed and maintained at Genentech in accordance with American Association of Laboratory Animal Care guidelines. All experimental studies were conducted under protocol 13-0979A and approved by the Institutional Animal Care and Use Committee of Genentech Lab Animal Research and performed in an Association for Assessment and Accreditation of Laboratory Animal Care International (AAALAC)-accredited facility in accordance with the Guide for the Care and Use of Laboratory Animals and applicable laws and regulations.

### Antibodies.

The anti-Pal antibody was a generous gift from Shaw Warren (Massachusetts General Hospital). The anti-OmpA (Antibody Research Corporation), anti-GroEL (Enzo Life Sciences), anti-ThyA (GeneTex, Inc.) and anti-His (Cell Signaling Technology) antibodies were obtained from commercial sources. Generation of anti-Lpp and anti-BamA antibodies was previously described (17, 60, 61). Recombinant Lgt was used to generate rabbit polyclonal antibodies. Rabbit immunizations, generation of antisera, and purification of rabbit polyclonal antibodies were performed as previously described for Lpp (17).

### Generation of bacterial strains and plasmids.

Bacterial strains and plasmids used in this study are listed in Table S1 in the supplemental material. E. coli strain CFT073 (ATCC 700928) (62) and MG1655 (ATCC 700926) were purchased from ATCC. Gene disruption in CFT073 was performed as previously described (17, 63). CFT073 Δ*lgt* and MG1655 Δ*lspA* were generated using λ Red recombination (17, 63). The primers used to generate the CFT073 and MG1655 mutants are listed in Table S2. Plasmids pKD46 for the λ Red recombinase (17, 63), pKD4 or pSim18 for the integration construction (17, 63), and pCP20 (64) for the FLP recombinase were used in this study. The 3′ primers (Table S2) used to generate the integration construct from pKD4 contain a juxtaposed ribosome binding site downstream of the kanamycin (Kan) cassette to allow for the creation of nonpolar gene deletions within operons (63), which is critical when generating the *lgt* inducible deletion strain given that the ribosomal binding site of the *thyA* gene downstream of *lgt* is within the *lgt* coding region. The inducible deletion strains (MG1655 Δ*lgt* and MG1655 Δ*lolCDE*) in either the WT and/or Δ*lpp* backgrounds were generated using similar methods as previously described (17, 65). To delete *lpp* in the *lgt*, *lspA*, and *lolCDE* inducible deletion strains, the Kan cassette was removed by transforming the FLP recombinase-expressing pCP20 plasmid followed by deletion of the *lpp* gene using λ Red recombinase as discussed above. Similar protocols were used to introduce the *imp4213* mutant allele. The PA14 *imp*4213 strain was generated based on published protocols (66–68). Briefly, the *lptD imp*4213 mutant allele was generated by PCR amplifying PA14 genomic DNA using primer pairs PA14*imp*4213_up-F/PA14*imp*4213_up-R (upstream region) and PA14*imp*4213_dn-F/PA14*imp*4213_dn-R (downstream region) to create *lptD* missing amino acids 425 to 447. PCR products were combined with the PCR-amplified vector pEX18gm with primers pLMG18_GA-F/pLMG18_GA-R and assembled using Gibson assembly (NEB). The resultant suicide vector pEX18gm was confirmed by sequence analysis (ELIM Biopharm). Single recombination mutants were selected on LB containing 10 μg/ml gentamicin and 25 μg/ml irgasan. Double recombination mutants were selected on LB without NaCl plates containing 10% sucrose and confirmed by PCR and sequence analysis. For expression under the isopropyl-β-d-thiogalactopyranoside (IPTG)-inducible promoter, DNA encoding the full-length sequences of *lgt*, *lspA*, *lnt*, and *lolCDE* were cloned into pLMG18 and induced using 2.5 mM IPTG.

### *In vitro* growth inhibition and serum sensitivity assays.

Unless stated otherwise, E. coli cells were grown in Luria-Bertani (LB) medium (0.5% yeast extract, 1% tryptone, 0.5% NaCl) at 37°C. Where indicated, kanamycin (Kan) was added to culture media at a 50-μg/ml final concentration. MIC assays were performed based on Clinical and Laboratory Standards Institute (CLSI) guidelines. For *in vitro* growth curves, overnight cultures of WT CFT073, CFT073 Δ*lgt*, and CFT073 Δ*lgt* complemented with *lgt* from E. coli (*lgt*^Ec^) or P. aeruginosa (*lgt*^Pa^) were grown to mid-exponential phase (optical density at 600 nm [OD_600_] of 0.6) and then diluted to an OD_600_ of 0.1 to initiate growth curves. At various times, culture aliquots were diluted and plated in dilutions on LB+Kan agar, and CFU were enumerated in duplicates. Growth of MG1655 inducible deletion strains was measured by culturing in the presence of 2-fold dilutions of arabinose (starting arabinose concentrations for CFT073 and MG1655 inducible deletion strains were 4% and 0.8%, respectively). While 2% arabinose was sufficient for WT growth of MG1655 Δ*lgt*, 4% arabinose was used for CFT073 Δ*lgt* based on comparing its growth to that of WT CFT073 as measured by CFU. OD_600_ growth measurements were performed using an EnVision 2101 multilabel reader plate reader (PerkinElmer) linked with Echo liquid handler (Labcyte). For time-kill experiments, bacteria were harvested in mid-exponential phase and treated with 12.5 μM G2824, 3.2 μM globomycin, and 1.6 μM vancomycin. CFU were enumerated at various times posttreatment. Bacterial culture medium containing 2% arabinose was used to induce *lpp* or *lpp*ΔK expression from pBad24 plasmids. Bacterial viability at different time points during the treatment was measured by enumerating CFU. Serum killing assays were carried out as previously described (17).

### Detection of membrane permeability using SYTOX green incorporation.

To determine the effect of Lgt depletion on membrane permeability, WT CFT073 and CFT073 Δ*lgt* strains were streaked onto LB agar plates containing 4% arabinose and cultured at 37°C for 18 h. From a single colony, bacteria were cultured in LB broth containing 4% arabinose and cultured at 37°C to an OD of 0.5. One-milliliter cultures at an OD of 0.5 for both strains were harvested, washed, and resuspended in LB broth or medium containing a range of arabinose (2, 0.2, 0.1, 0.05, and 0%) or glucose (0.2%) concentrations and incubated at 37°C for 2 h. Cells were harvested by centrifugation at 4,000 × *g* at 4°C for 5 min. Intact CFT073 or CFT073 treated with 70% ethanol at room temperature (RT) for 15 min to permeabilize the cells were used as controls. Cells were incubated with SYTOX green according to the manufacturer’s recommendations, washed with phosphate-buffered saline (PBS; 3 times) and fixed in 2% paraformaldehyde. SYTOX green incorporation was measured by flow cytometry using a FACSAria II (Becton Dickenson) and analyzed using FlowJo software.

### Mouse infections.

Overnight bacterial cultures were back diluted 1:100 in M9 medium and grown to an OD_600_ of 0.8 to 1 at 37°C. Cells were harvested, washed once with PBS, and resuspended in PBS containing 10% glycerol. Cells were frozen in aliquots, and thawed aliquots were measured for CFU prior to mouse infections. Virulence of WT CFT073 and CFT073 Δ*lgt* was measured using the neutropenic E. coli infection model (69). Seven-week-old female A/J mice (Jackson Laboratory) were rendered neutropenic by peritoneal injections of 2 doses of cyclophosphamide (150 mg/kg body weight on day −4 and 100 mg/kg body weight on day −1). On day 0, mice were infected with 5 × 10^5^ CFU of mid-exponential-phase bacteria diluted in PBS by intravenous injection through the tail vein. At 30 min and 24 h postinfection, bacterial burdens in the liver and spleen were determined by serial dilutions of tissue homogenates on LB plates.

### Expression and purification of recombinant Lgt.

DNA encoding full-length E. coli Lgt fused to a C-terminal Flag tag was transformed into Rosetta 2(DE3) Gold cells (Agilent). Starter cultures were grown in Terrific broth (TB) medium with carbenicillin (50 μg/ml) and chloramphenicol (12.5 μg/ml) at 37°C for 3 h. The starter cultures were diluted 1:50 in TB medium with carbenicillin (50 μg/ml), chloramphenicol (12.5 μg/ml), and glycerol (1%) and grown at 37°C for 2 h with shaking at 200 rpm. The temperature of the culture was reduced to 30°C, and they were grown for an additional 2 h before the temperature of the culture was reduced to 16°C for growth for 64 h. The cells were harvested by centrifugation, resuspended in lysis buffer (20 mM Tris [pH 8.0], 300 mM NaCl, protease inhibitor cocktail, and Lysonase), and stirred at 4°C for 30 min before being passed through a microfluidizer 3 times. The membrane fraction was solubilized by adding DDM directly to the lysate to a final concentration of 1% and stirring at 4°C for 2 h before centrifugation at 40,000 rpm for 1 h. Preequilibrated Flag resin was added to the supernatant and incubated with rotation at 4°C for 2 h. The slurry was added to a gravity column, and the column was washed with 10 culture volumes (CV) buffer A (20 mM Tris [pH 8.0], 300 mM NaCl, 5% glycerol, 1% DDM) and 10 CV buffer B (20 mM Tris [pH 8.0], 300 mM NaCl, 5% glycerol, 0.05% DDM). The bound fraction was eluted by the addition of 5 CV of buffer C (20 mM Tris [pH 8.0], 300 mM NaCl, 5% glycerol, 0.05% DDM, 100 μg/ml Flag peptide). The peak fractions were collected, concentrated to less than 5 ml, and loaded onto a Superdex 200 16/60 column equilibrated with buffer C [20 mM Tris (pH 8.0), 300 mM NaCl, 5% glycerol, 0.05% DDM, 1 mM Tris(2-carboxyethyl)phosphine hydrochloride (TCEP)]. The peak fractions were collected, analyzed by SDS-PAGE, and stored at −80°C.

### Macrocyclic peptide library design and selection of Lgt-binding molecules.

A thioether-macrocyclic peptide library was constructed by using *N*-chloroacetyl d-phenylalanine (ClAc-f) as an initiator in a genetically reprogrammed *in vitro* translation system (36). The genetic code was designed with the addition of two *N*-methyl amino acids, *N*-methyl-l-phenylalanine (MeF) and *N*-methyl-l-glycine (MeG), and three unnatural amino acids, (*S*)-2-aminoheptanoic acid (Ahp), (*S*)-3-([1,1′-biphenyl]-4-yl)-2-aminopropanoic acid (Bph), and (*S*)-1,2,3,4-tetrahydroisoquinoline-3-carboxylic acid (Tic) in addition to 11 natural amino acids (Ser, Tyr, Trp, Leu, Pro, His, Arg, Asn, Val, Asp, and Gly). After *in vitro* translation, a thioether bond formed spontaneously between the N-terminal ClAc group of the initiator d-phenylalanine residue and the sulfhydryl group of a downstream cysteine residue to generate the macrocyclic peptides.

Lgt was biotinylated by the addition of 400 μl of a freshly prepared solution of 20 mM *N*-hydroxysuccinimide (NHS)-polyethylene glycol 4 (PEG 4)-biotin (Thermo) to 1.375 ml of 10 mg/ml Lgt, as per the manufacturer’s instructions. The reaction mixture was incubated for 30 min at RT, and free NHS-PEG 4-biotin was removed by gel filtration chromatography on a Superdex 200 16/60 column that had been preequilibrated with 20 mM Tris (pH 8.0), 250 mM NaCl, 5% glycerol, and 0.02% DDM. The final biotinylated sample was concentrated to 1 mg/ml. Affinity selection of macrocyclic peptides binding to Lgt was performed using E. coli Lgt-biotin in 0.02% *n*-dodecyl β-d-maltoside (DDM). Briefly, 10 μM mRNA library was hybridized with a peptide linker (11 μM) at RT for 3 min. The mRNA library was translated at 37°C for 30 min in the reprogrammed *in vitro* translation system to generate the peptide-mRNA fusion library (35, 37). Each reaction mixture contained 2 μM mRNA-peptide-linker conjugate, 12.5 μM initiator tRNA (tRNAfMet aminoacylated with ClAc-d-Phe), and 25 μM each elongator tRNA aminoacylated with the specified noncanonical/canonical amino acids. In the first round of selections, translation was performed at a 20-μl scale. After the translation, the reaction was quenched with 17 mM EDTA. The product was subsequently reverse transcribed using RNase H minus reverse transcriptase (Promega) at 42°C for 30 min, and buffer was exchanged for DDM buffer: 50 mM Tris (pH 8), 5 mM EDTA, 200 mM NaCl, 0.02% DDM, and 1 mM glutathione. For affinity selection, the peptide-mRNA/cDNA solution was incubated with 250 nM biotinylated E. coli Lgt for 60 min at 4°C, and the streptavidin-coated beads (Dynabeads M-280 streptavidin; Thermo) were further added and incubated for 10 min to isolate Lgt binders. The beads were washed once with cold DDM buffer, the cDNA was eluted from the beads by heating for 5 min at 95°C, and fractional recovery from the affinity selection step was assessed by quantitative PCR using Sybr green I on a LightCycler thermal cycler (Roche). After five rounds of affinity maturation, two additional rounds of off-rate selections were performed by increasing the wash stringency before elution to identify high-affinity binders. Sequencing of the final enriched cDNA was carried out using a MiSeq next-generation sequencer (Illumina).

### Peptide synthesis.

Thioether macrocyclic peptides were synthesized using standard 9-fluorenylmethoxy carbonyl (Fmoc) solid-phase peptide synthesis (SPPS). Following coupling of all amino acids, the deprotected N terminus was chloroacetylated on resin followed by global deprotection using a trifluoroacetic acid (TFA) deprotection cocktail. The peptides were then precipitated from the deprotection solution by adding >10-fold excess diethyl ether. Crude peptide pellets were then dissolved and repelleted 3 times using diethyl ether. After the final wash, the pellet was left to dry, and then the pellet was resuspended in dimethyl sulfoxide (DMSO) followed by the addition of triethylamine for intramolecular cyclization via formation of a thioether bond between the thiol of the cysteine and N-terminal chloroacetyl group. Upon completion of cyclization, the reaction was quenched with acetic acid (AcOH), and the cyclic peptide was purified using standard reverse-phase high-pressure liquid chromatography (HPLC) methods. For the Lgt biochemical assay, the Pal-IAAC and Pal-IAAA peptides were synthesized by CPC Scientific.

### SDS-PAGE and Western immunoblotting.

Bacterial cell samples normalized for equivalent OD_600_ and resuspended in BugBuster lysis buffer (Fischer Scientific) with the addition of sample buffer (LI-COR), and proteins were separated by SDS-PAGE using 16% Tricine protein gels or NuPAGE 4% to 20% bis-Tris gels (Thermo Fisher Scientific), transferred to nitrocellulose membranes using the iBlot 2 dry blotting system (Invitrogen), and blocked using LI-COR blocking buffer for 30 min. Unless stated otherwise, loading buffer with reducing agents was added and samples were not boiled prior to SDS-PAGE. For the sucrose gradient centrifugation, samples were boiled prior to running the SDS-PAGE. Primary antibodies were used at a final concentration of 1 μg/ml with some exceptions: rabbit anti-Lpp polyclonal antibody (0.1 μg/ml), murine anti-Pal 6D7 antibody (0.5 μg/ml), rabbit anti-GroEL antibody (1:10,000 final dilution), and rabbit anti-OmpA antibody (1:50,000 final dilution). The secondary antibodies were all obtained from LI-COR and used as per manufacturer’s instructions. Images were collected using the Odyssey CLx imaging system (LI-COR) and analyzed by Image Studio Lite.

### Development of the Lgt biochemical assay.

The Lgt enzymatic activity was measured by specific detection of G3P. Both G3P and G1P are released from phosphatidylglycerol as Lgt catalyzes the transfer of diacylglyceryl from phosphatidylglycerol to the preprolipoprotein substrate, since the PGN substrate used in the assay contains a racemic glycerol moiety at the end of phosphatidyl group. The standard assay consists of a 6-μl reaction mixture with 3 nM Lgt-DDM, 50 μM phosphatidylglycerol (1,2-dipalmitoyl-*sn*-glycero-3-phospho-[1′-*rac*-glycerol]; Avanti), and 12.5 μM Pal-IAAC peptide substrate derived from the Pal lipoprotein (MQLNKVLKGLMIALPVMAIAACSSNKN) in 50 mM Tris (pH 8), 200 mM NaCl, 5 mM EDTA, 0.02% DDM, 0.05% bovine skin gelatin, and 1 mM glutathione. As a control, we used a mutant nonmodifiable Pal substrate peptide containing a cysteine-to-alanine mutation (Pal-IAAA), which served as a competitive nonmodifiable inhibitor. The reaction was quenched after 60 min at RT with 0.5 μl of 4.8% lauryl dimethylamine-*N*-oxide (Anatrace), followed by addition of 6 μl detection solution. After incubation for 120 min at RT, the luminescence signal was read. The detection solution was modified based on an NAD Glo protocol (Promega, G9072), per the manufacturer’s instructions. Specifically, 10 ml detection solution consists of 3-fold dilution of luciferin detection reagents, supplemented with 10 μl reductase, 2.5 μl reductase substrate, 1 mM NAD, and 4.25 U of glycerol-3-phosphate dehydrogenase (G3PDH) (Roche Diagnostics, 10127779001). The luciferin detection reagents, reductase, and reductase substrate were all from the NAD Glo kit (Promega). Luminescence values were normalized to those of DMSO controls (0% inhibition) and no enzyme controls (100% inhibition). IC_50_ values were calculated using a 4-parameter logistic model using GraphPad Prism software.

### Mass spectrometry.

Ammonium acetate, acetic acid, formic acid, and trifluoroacetic acid were purchased from Sigma-Aldrich (St. Louis, MO). Acetonitrile (ACN) was purchased from Fisher Scientific (Hampton, NH).

For liquid chromatography UV mass spectrometry (LC-UV-MS) analysis and top down sequencing, 1 μl corresponding to 12.5 μM Pal peptide, MQLNKVLKGLMIALPVMAIAACSSNKN (2859.6 Da), was injected onto a Q Exactive UHMR Orbitrap (70) coupled to a Vanquish liquid chromatograph (Thermo Fisher Scientific) through a 2.1 mm by 50 mm MAbPac reverse-phase (RP) HPLC column (80°C). Ions of interest, representing the most abundant charge state of the peptide with or without Lgt treatment, *m/z* 953.86 (3+) and 1,137.69 (3+), respectively, were isolated in the quadrupole and selected for fragmentation in the high-energy collisional dissociation (HCD) cell at a gas pressure of 3 × 10^−5^ to 7 × 10^−5 ^Pa, with UHMR mode “on.” The MS parameters used were spray voltage of 1.2 to 1.3 V, source temperature of 300°C, collision energy of 60 V, source dissociation energy of 5 V, and resolution (at *m/z* 200) of 35,000. The instrument was mass calibrated as described previously using a solution of CsI (71).

Theoretical fragment ions were generated using MS-Product in Protein Prospector (https://prospector.ucsf.edu) and annotated manually to determine the exact mass addition on Cys. Spectra were subsequently analyzed and fragmentation map generated in ProSightLite (http://prosightlite.northwestern.edu) with the assigned mass addition to Cys for the treated peptide. Fragment ions were matched to within 10 ppm of theoretical *b*-type and *y*-type ions.

### Transmission electron microscopy, time-lapse microcopy, and confocal microcopy.

Electron microscopy was performed as previously described (65). For time-lapse microscopy, WT CFT073, CFT073 Δ*lgt*, and CFT073 Δ*lgt* Δ*lpp* cells were grown overnight in LB medium containing 4% arabinose, back diluted to a final OD_600_ of 0.1, and immediately placed between a coverslip and 1% agarose pad containing 0.2% glucose for imaging. Cells were maintained at 37°C during imaging in a stage-top chamber (Okolab Inc.). Cells were imaged on a Nikon Eclipse Ti inverted confocal microscope (Nikon Instruments Inc.) coupled with an UltraVIEW VoX (PerkinElmer Inc.) and a 100× (numerical aperture [NA], 1.40) oil immersion lens objective. Images were captured at various times using an ORCA-Flash 4.0 complementary metal oxide semiconductor (CMOS) camera (Hamamatsu Photonics), collected using Volocity software (Quorum Technologies), and processed using Fiji (72). For confocal microscopy, images were acquired on a Leica SP8 STED 3× platform using a 100× white-light, NA 1.4 oil immersion lens objective. Cells were treated with arabinose or glucose (CFT073 Δ*lgt*) or G2824 or globomycin at 1× MIC for 30 min (CFT073 *imp4213*), fixed with 4% paraformaldehyde, and incubated with 1 μg/ml FM-64 dye and 1 μg/ml 4′,6-diamidino-2-phenylindole (DAPI) solution. Quantitation of bacterial cell area was performed using the ImageJ program by measuring at least ∼100 bacterial cells from two independent experiments.

### Downregulation of gene expression by CRISPRi.

The two-plasmid bacterial CRISPRi system pdCas9-bacteria_GNE and PGNRNA-bacteria_GNE are based on the AddGene plasmids 44249 and 44251 (73), respectively. The plasmid was synthesized in smaller DNA fragments (500 bp to 3 kb) (IDT gBlocks) and assembled by Gibson assembly (NEB) according to the manufacturer’s protocols. Plasmids were confirmed by sequencing (ELIM Bio). gRNAs were designed to target the 5′ end of the gene on the nontemplate strand using Benchling CRISPR software (74). gRNAs were cloned into PGNRNA-bacteria using Gibson assembly (NEB) according to the manufacturer’s protocols and sequence confirmed (ELIM Bio).

Bacterial cultures were grown overnight on LB agar supplemented with carbenicillin (50 μg/ml) and chloramphenicol (12.5 μg/ml) to maintain both plasmids, pdCas9-bacteria and PGNRNA-bacteria with each gRNA as appropriate. Cells were scraped from the plate into fresh medium. The OD_600_ was measured, and the culture was subsequently diluted to an OD_600_ 0.001 in the presence or absence of G2824, globomycin, or ciprofloxacin. Two hundred microliters was transferred to a 96-well plate (Corning) and monitored for growth by measuring the OD_600_ (EnVision multimode plate reader; PerkinElmer). All treatments were performed in triplicates. The specificity of CRISPRi downregulation was measured using reverse transcriptase quantitative PCR (RT-qPCR).

### Purification of peptidoglycan-associated proteins.

Purification of PGN-associated proteins (PAP) was performed according to published methods (17, 40, 41) with some modifications. Briefly, bacteria were harvested in mid-exponential phase for treatment and then subjected to PAP extraction by resuspending the cell pellets from an OD (*A*_600_) of 10 in 6 ml of PAP extraction buffer containing 2% (wt/vol) SDS in 100 mM Tris-HCl (pH 8.0) with 100 mM NaCl, 10% glycerol, and cOmplete, mini, EDTA-free protease inhibitor cocktail (Sigma-Aldrich). After 60 min at RT, the extraction was subjected to centrifugation at 100,000 × *g* for 60 min at 22°C, and the pellet, containing PGN and associated proteins, was washed once with the same PAP extraction buffer with centrifugation at 100,000 × *g* for 30 min and resuspended in 200 μl of PAP extraction buffer (referred to as the SDS-insoluble on PAP fraction). The supernatant containing the SDS-soluble fraction was aliquoted and frozen (referred to as non-PAP fraction). Both fractions were treated with equal volumes of BugBuster buffer prior to the addition of sample buffer for Western immunoblotting as described above. It should be noted that the final PAP fractions were ∼30-fold more concentrated than the non-PAP fractions.

### Isolation of E. coli IM and OM using sucrose gradient centrifugation.

Bacterial inner and outer membranes were separated by isopycnic sucrose gradient centrifugation as described previously (75) and by the Hancock Laboratory (http://cmdr.ubc.ca/bobh/method/outer-membrane-preparation-one-step-sucrose-gradient-procedure/), with some modifications. Briefly, bacteria were grown in Luria broth at 37°C to mid-exponential phase (OD_600_ of 0.8 to 0.9) and then treated with 1× MIC of designated inhibitors for 1 h. Cells representing equivalents of an OD_600_ of 10 were harvested by centrifugation at 4,000 × *g* for 15 min, washed once, and resuspended in cold 10 mM Tris, pH 8.0, containing 20% (wt/vol) sucrose, cOmplete EDTA-free protease inhibitor cocktail (Roche), and 1,000 U/ml nuclease (Benzonase nuclease; EMD Millipore). The cell suspension was disrupted by two passages through an LV1 Microfluidizer (Microfluidics). Unbroken cells were removed by centrifugation at 4,000 × *g*, and membranes were collected by ultracentrifugation at 100,000 × *g* for 1 h and washed once with cold 10 mM Tris, pH 8.0. The final membrane preparation was resuspended in 10 mM Tris, pH 8.0, containing 10% sucrose, cOmplete EDTA-free protease inhibitor cocktail (Roche), and 1,000 U/ml nuclease and then applied to the top of the sucrose gradients (73%, 60%, and 20%). The loaded gradients were spun at 39,000 rpm for 22 h at 4°C in a Beckman SW 41 Ti rotor. Fractions were collected from the top and analyzed by SDS-PAGE and immunoblotting with appropriate antibodies.

### Statistical analyses.

All statistical analyses were performed using GraphPad Prism 6.0 software. The data were tested to ensure appropriate use in parametric statistics, and statistical analyses were performed on log-transformed data. All graphs present the means ± the standard errors of the means (SEMs). Unless stated otherwise, *P* values for all data were determined using a Mann-Whitney unpaired *t* test. Bonferroni’s correction was applied to control for multiple comparisons for CRIPSRi data shown in Fig. 4G.
