# Supplementary material for: Inhibition of Escherichia coli Lipoprotein Diacylglyceryl Transferase Is Insensitive to Resistance Caused by Deletion of Braun’s Lipoprotein
Source: J Bacteriol. 2021 Jun 8;203(13):e00149-21. doi: 10.1128/JB.00149-21 (PMC8316002; doi:10.1128/JB.00149-21)
Supplement: Supplemental file 1 — Fig. S1 to S5 and Tables S1 and S2. Download JB.00149-21-s0001.pdf, PDF file, 1.11 MB [file jb.00149-21-s0001.pdf]

Supplemental Figures and Tables

Figure S1

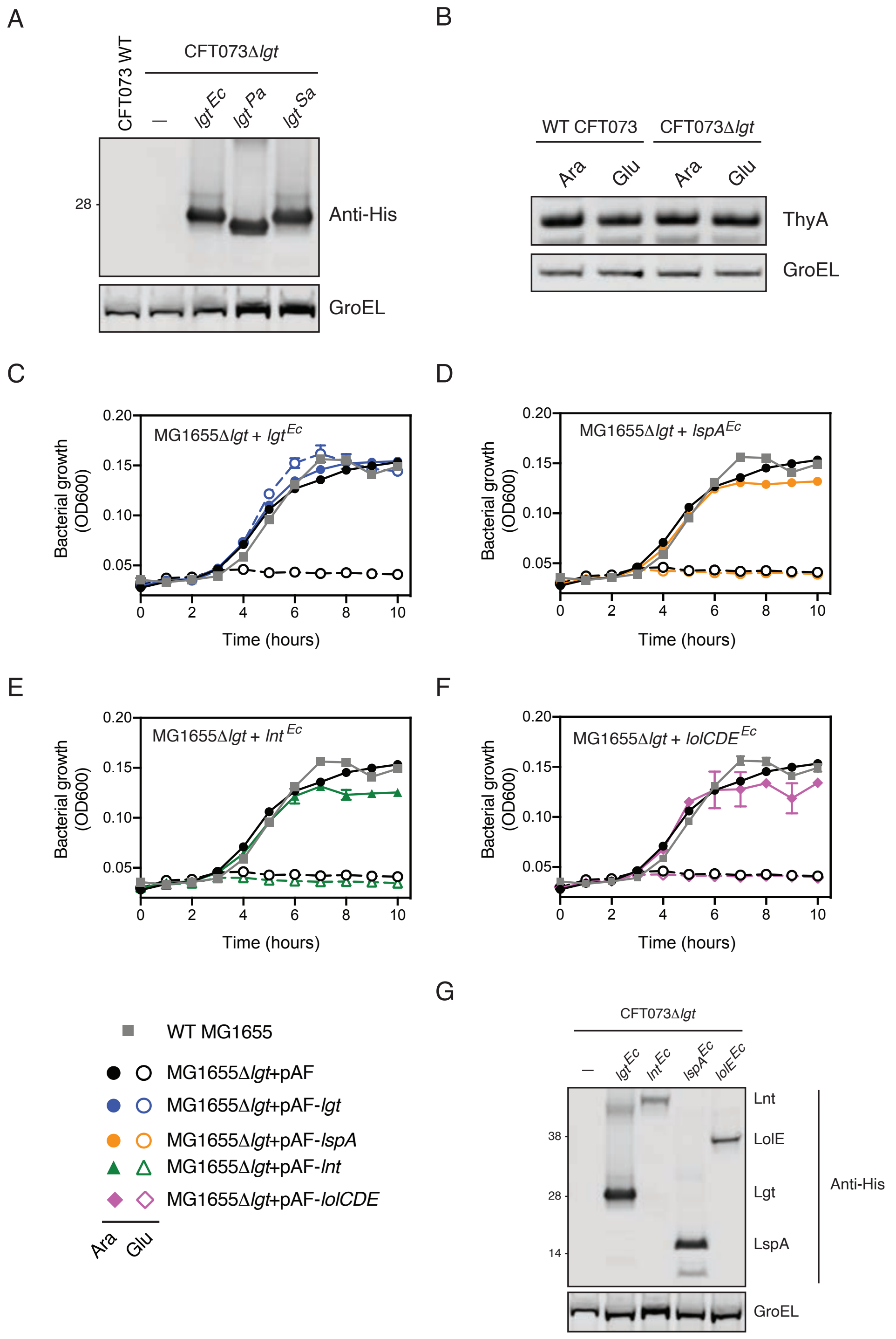

Figure S1: (A) Western blot analyses confirming protein expression after complementation with pLMG18 expressing *lgt* from *E. coli* (*lgt*^{Ec}), *P. aeruginosa* (*lgt*^{Pa}) or *S. aureus* (*lgt*^{Sa}). All complemented *lpp* contain a c-terminal His-tag. **(B)** Expression of thymidylate synthase (ThyA) is not affected after depletion of Lgt. WT CFT073 and CFT073Δ*lgt* were grown under wild-type (4% arabinose, Ara) or depleted (0.2% glucose, Glu) conditions for 4 hours and total cell lysates were subjected to Western blot analyses using an anti-ThyA antibody. GroEL was used as a loading control. **(C-F)** Loss of *E. coli* MG1655Δ*lgt* viability after Lgt depletion is rescued after complementing with *E. coli lgt* (*lgt*^{Ec}) but not *E. coli lspA* (*lspA*^{Ec}), *lnt* (*lnt*^{Ec}) or *lolCDE* (*lolCDE*^{Ec}). 2.5 mM IPTG was used to induce expression of *E. coli lspA*, *lnt* or *lolCDE*. Cells were grown in arabinose (filled symbols, Ara) or glucose (open symbols, Glu) and bacterial growth was measured by OD_{600}. **(G)** Anti-His Western blot analyses demonstrating protein expression of *E. coli* Lgt, LspA, Lnt and LolE in CFT073Δ*lgt* cells complemented with His-tagged versions of the respective genes.

Figure S2

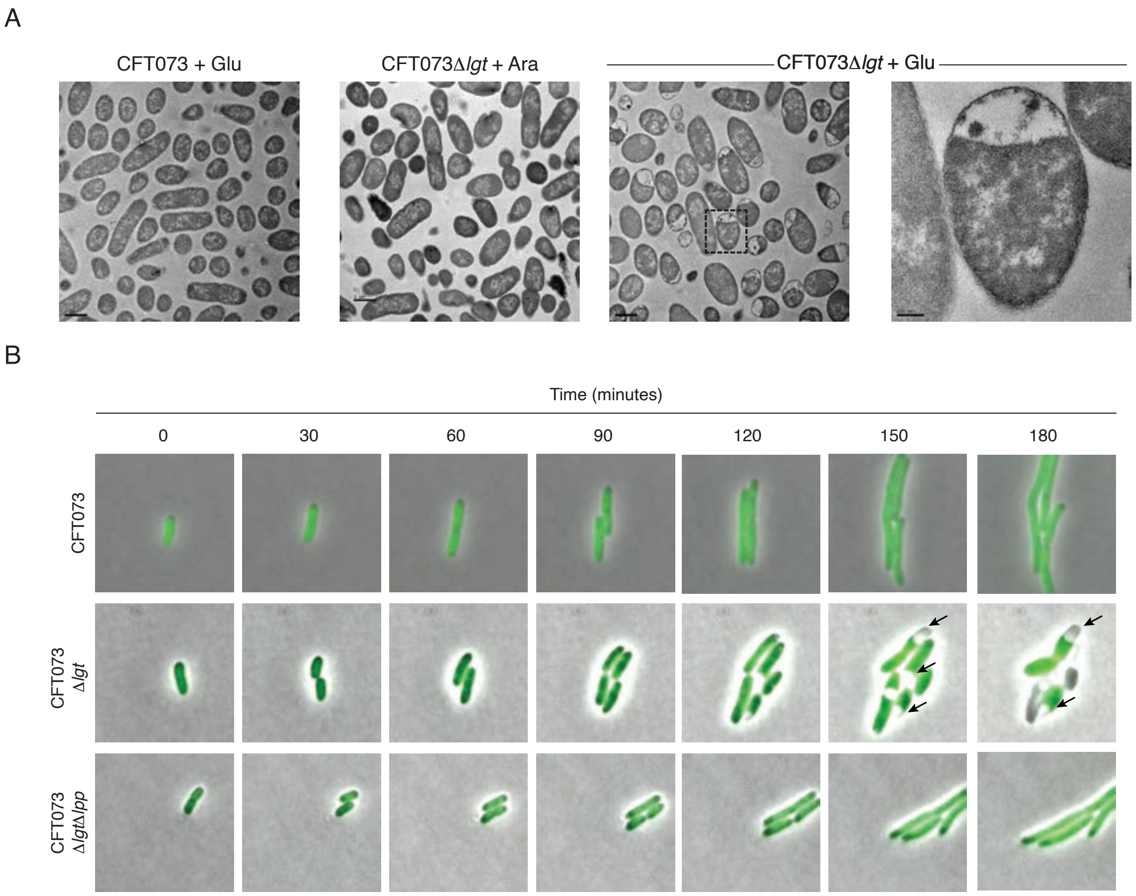

Figure S2: Lgt depletion results in IM contraction and the expected globular cellular phenotype. **(A)** CFT073 and CFT073Δ*lgt* deletion strains were treated for 2 hours with 4% arabinose (Ara) or 0.2% glucose (Glu) and samples were processed for imaging by Transmission electron microscopy. Bars represent 1 μm (200 nm for last panel). **(B)** Live cell imaging of WT CFT073, CFT073Δ*lgt* and CFT073Δ*lgt*Δ*lpp* inducible deletion strains containing a plasmid expressing *gfp* (pGFP) were grown in the presence of 0.2% glucose. Phase contrast and fluorescence microscopy images were overlayed at various times post treatment. Arrows denote IM contraction which is not observed in the strain containing the *lpp* deletion.

Figure S3

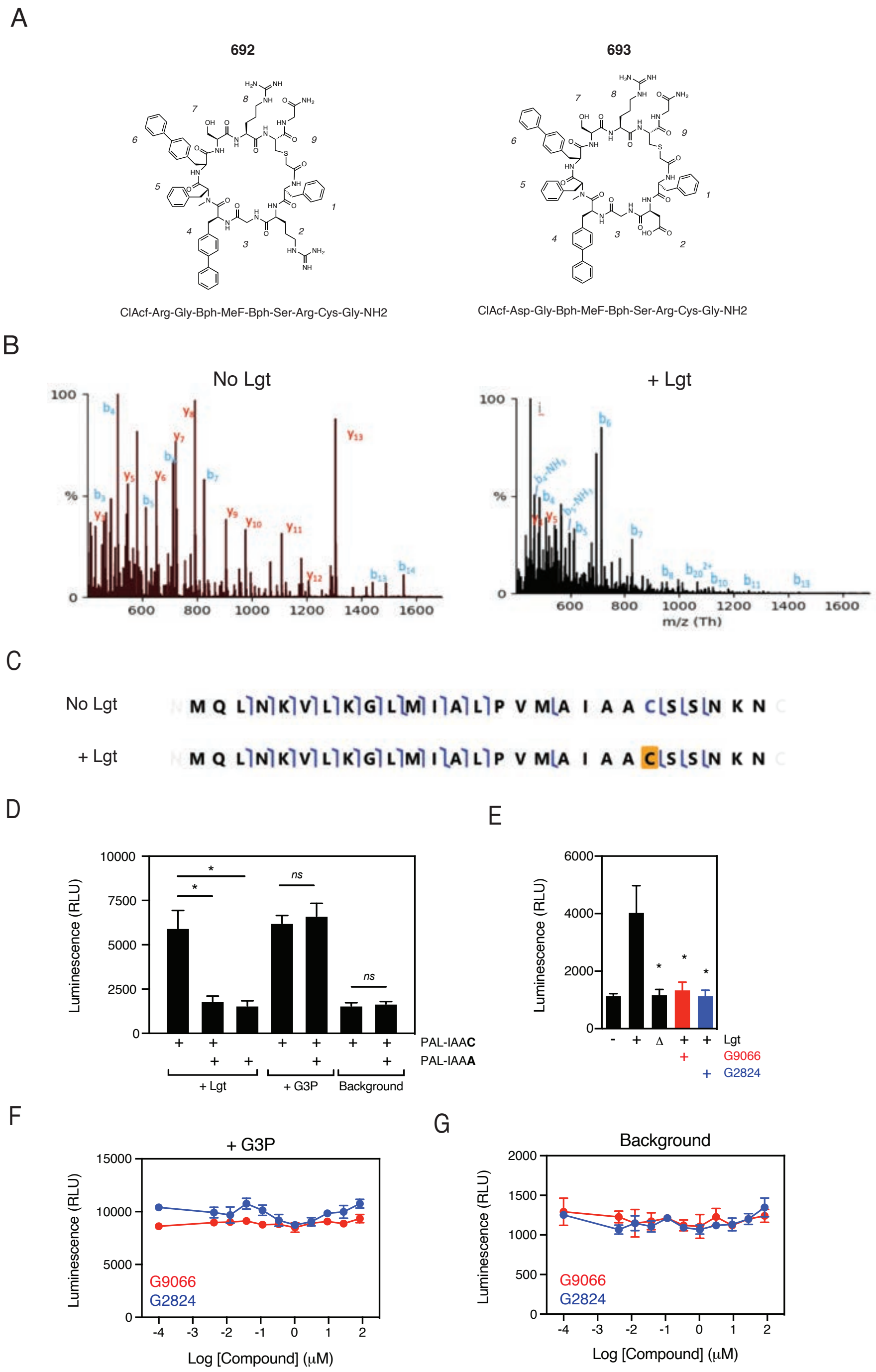

Figure S3: (A) Chemical structures of original hit macrocycles **692** and **693** identified in the library

screen. The sequences of the macrocycles are represented in a linear format using the three letter amino acid codes. Non-natural amino acids are as follows: N-α-Methyl-L-phenylalanine (MeF), and 4-Phenyl-L-phenylalanine (Bph). ClAcf was fixed at the first position and used for cyclization. **(B)** MS/MS spectra of the Pal-IAA**C** peptide. Lgt reactions were performed using 50 μM phosphatidylglycerol and 38 μM Pal-IAA**C** peptide in the absence (m/z 953.86, 3+, No Lgt) or presence (m/z 1137.69, 3+, + Lgt) of 100 nM Lgt. **(C)** Sequence coverage of the Pal-IAA**C** peptide -/+ Lgt. **(D)** Standard Lgt reactions were performed with 12.5 μM Pal-IAA**C**, 75 μM Pal-IAA**A**, or 12.5 μM Pal-IAA**C** + 75 μM Pal-IAA**A**. To rule out that Pal-IAA**A** had non-specific effects on the coupling reaction, Lgt reaction components were replaced with 0.6 μM G3P (+G3P) and incubated with either 12.5 μM Pal-IAA**C** or 12.5 μM Pal-IAA**C** + 75 μM Pal-IAA**A**. To rule out any effects on the background signal of the reaction, Lgt reaction mixture with no Lgt present were treated, as described above. Data are representative of experiments performed in triplicate (*ns* = not significant, *p = 0.0122, 0.0138). **(E)** Heat inactivation of Lgt leads to loss of enzymatic activity. Standard Lgt reactions were performed using Lgt that was left untreated or heat inactivated (Δ) at 98°C for 10 minutes. As controls, Lgt reactions were performed with or without 20 μM G9066 or G2824 (*p = 0.0293, 0.0304, 0.0301). **(F, G)** Product control (1.2 μM G3P) **(F)** and background (no Lgt) **(G)** samples were incubated with either G9066 or G2824 at the indicated concentrations to confirm lack of non-specific effects on the reagents present in the coupling reaction. Data are representative of experiments performed in triplicate.

Figure S4

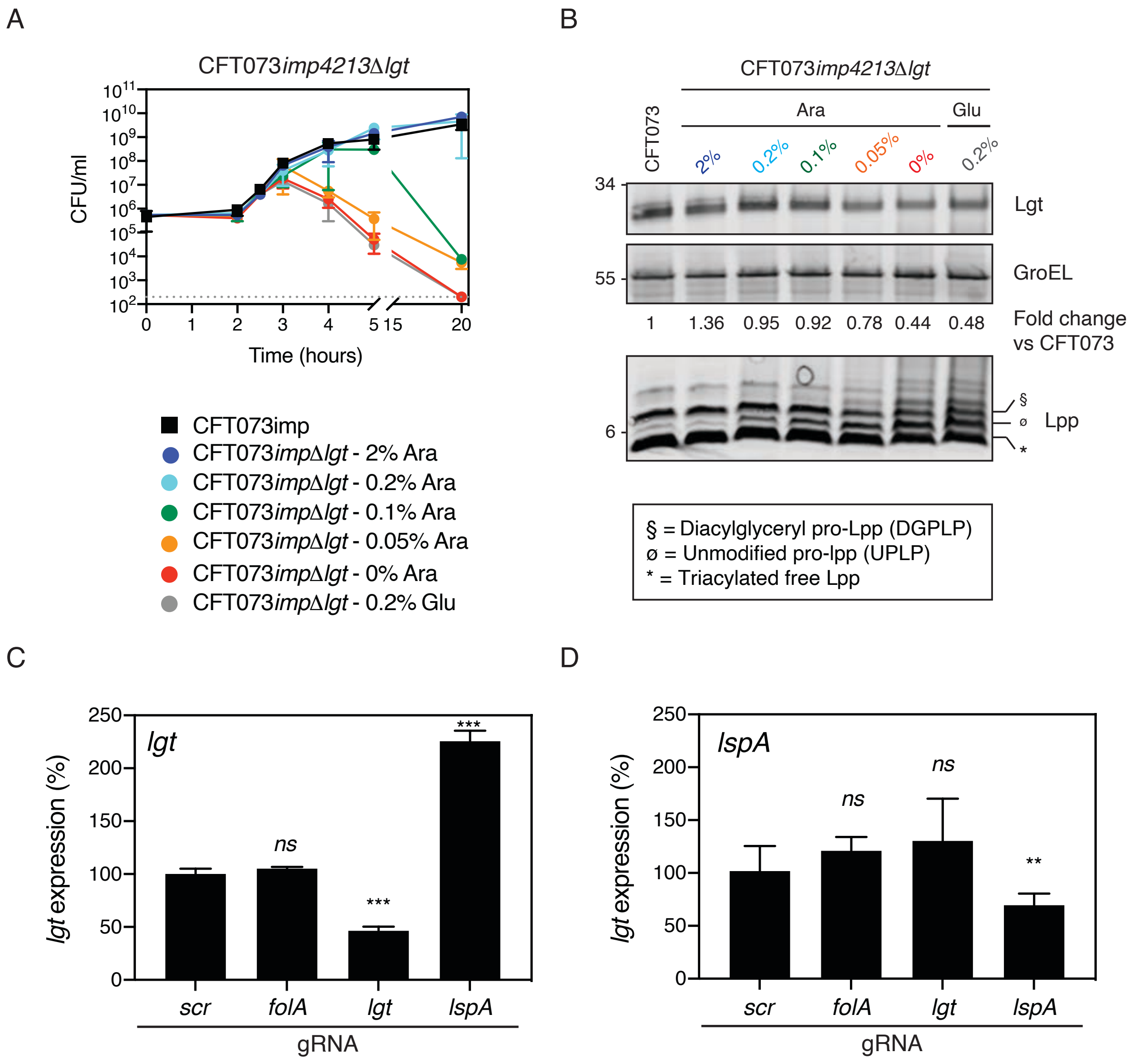

Figure S4: (A) CFT073*im*p4213Δ*lgt* cells were treated with a range of arabinose concentrations and CFUs were enumerated over 20 hours. CFU growth data are representative of two independent experiments performed in duplicate. **(B)** Western blot analysis for expression of Lgt and Lpp was performed using total cell lysates from untreated WT CFT073 or CFT073*imp*4213Δ*lgt* treated with arabinose or glucose for 3 hours. To quantitate Lgt expression levels, Lgt levels were normalized to

GroEL and quantitated as fold change relative to WT CFT073 (Fold change vs WT). Lpp forms are denoted as follows: * = triacylated free Lpp; § = PGN-linked diacylglyceryl pro-Lpp (DGPLP); ø = unmodified pro-Lpp (UPLP). Data are representative of two independent experiments. **(C-D)** Efficiency of CRISPRi-mediated downregulation of target genes. Total RNA was harvested from *E. coli* BW25113 cells transformed with scrambled (scr) gRNA or gRNA specific for *folA*, *lgt* and *lspA* and gene expression of *lgt* **(C)** and *lspA* **(D)** was measured by RT-qPCR. Relative gene expression of *lgt* and *lspA* were calculated by normalizing to *rpoB* levels using the $2^{-\Delta\Delta CT}$ method. Expression levels are graphed after comparison to "scr" gRNA, which was set at 100%. Data are representative of two independent experiments each performed in duplicate (*ns* = not significant, *p < 0.05, **p < 0.01, ***p < 0.001).

Figure S5

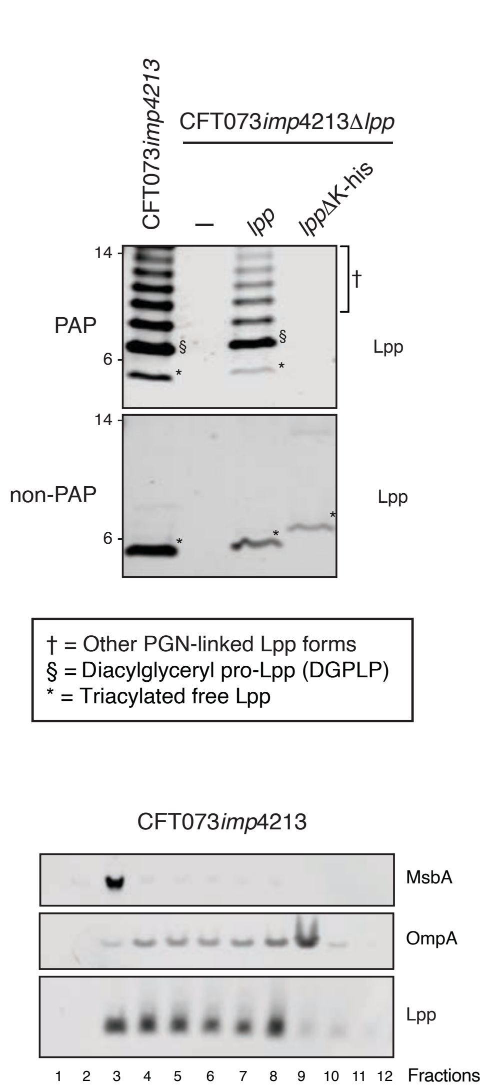

Figure S5: (A) Determination of PGN-linkage of WT Lpp and LppΔK. CFT073*imp*4213, CFT073*imp*4213Δ*lpp* or CFT073*imp*4213Δ*lpp* complemented with WT *lpp* or *lpp*ΔK were treated with SDS to isolate PAP and non-PAP fractions. Lpp levels were detected by Western blot analyses in total cell lysates, PAP and non-PAP fractions. The *lpp*ΔK construct is His-tagged and hence

migrates slower on SDS-PAGE compared to the free triacylated WT Lpp. Lpp forms are denoted in the figure (* = triacylated free Lpp; § = PGN-linked DGPLP; ø = UPLP; † = other PGN-linked Lpp forms; Δ = putative Lpp dimer)**. (B)** Untreated CFT0735*imp*4213 cells were subjected to isopycnic sucrose gradient ultracentrifugation. Fractions were loaded on SDS-PAGE and probed for MsbA, OmpA and Lpp by Western blot analysis.

Table S1: Bacterial strains and plasmids used in this study

Bacterial strains	Description	Reference
E. coli		
BW25113	rrnB3 DElacZ4787 hsdR514 DE(araBAD)567 DE(rhaBAD)568 rph-1	(1)
MG1655	*E. coli* K-12 F- lambda- *ilvG* negative, *rfb-50 rph-1*	ATCC 700926
MG1655Δ*lgt*	MG1655 Δ*lgt*::kan with an arabinose-inducible integrated *lgt* copy	This study
MG1655Δ*lgt*Δ*lpp*	MG1655 Δ*lpp*, Δ*lgt*::kan containing an arabinose-inducible integrated *lgt* copy	This study
MG1655Δ*lspA*	Arabinose-inducible conditional knockout of *lspA*	This study
MG1655Δ*lspA*Δ*lpp*	MG1655 Δ*lpp*, Δ*lspA*::kan containing an arabinose-inducible integrated *lspA* copy	This study
MG1655Δ*lolCDE*	Arabinose-inducible conditional knockout of *lolCDE*	This study
MG1655Δ*lolCDE*Δ*lpp*	MG1655 Δ*lpp*, Δ*lolCDE*::kan containing an arabinose-inducible integrated *lolCDE* copy	This study
CFT073	Bacteremia isolate, wild-type (O6:K2:H1)	ATCC 700928
CFT073Δ*lgt*	CFT073 Δ*lgt*::kan containing an arabinose-inducible integrated *lgt* copy	This study
CFT073*imp4213*	CFT073 carrying the *imp4213* allele in *lptD*	(2)
CFT073*imp4213*Δ*lgt*	CFT073 Δ*lgt*::kan containing an arabinose-inducible integrated *lgt* copy and carrying the *imp4213* allele in *lptD*	This study
CFT073*imp4213*Δ*lpp*	CFT073 Δ*lpp*::kan carrying the *imp4213* allele in *lptD*	This study
CFT073*imp*4213lpp^{Ara}	*CFT073imp*4213Δ*lpp*::kan containing pBAD24 expressing *lpp*	This study
CFT073*imp*4213$lpp\Delta K^{Ara}$	*CFT073imp*4213Δ*lpp*::kan containing pBAD24 expressing *lpp*Δ*K*	This study
TOP10	pWQ601, general cloning strain	Invitrogen
S. aureus USA300	USA300 FPR3757	Center for Staphylococcal Research, Nebraska
A. baumannii 19606	*Acinetobacter baumannii* strain isolated in a patient urine sample	ATCC
P. aeruginosa PA14	*Pseudomonas aeruginosa* strain UCBPP-PA14 originally isolated from a burn wound	ATCC
PA14*imp*4213	*Pseudomonas aeruginosa* UCBPP-PA14 containing the imp4213 mutation in *lptD*	This study
**Plasmids**		
pKD4	Kanamycin resistance (Kan^{R}) cassette flanked by FRT (FLP recognition target) sites, oriRγ	(3)
pKD46	Expresses the phage λ Red recombinase, Amp^{R}, temperature sensitive, oriRγ	(3)
pCP20	Thermal induction of FLP recombinase expression, Amp^{R}, temperature sensitive	(4)

pLDR8	Lambda integrase expression vector	ATCC 77357
pLDR9	Lambda att site integration vector	ATCC 77358
pBAD24	Arabinose inducible expression vector	ATCC 87399
pBAD24-*lpp*	pBad24 expressing *E. coli lpp*	This study
pBAD24-$lpp\Delta K$	pBad24 expressing *E. coli* $lpp\Delta K$	This study
pdCas9-bacteria_GNE	Based on AddGene plasmid 44249	This study
pgRNA-bacteria_GNE	Based on AddGene plasmid 44251	This study
pLMG18	Low-copy IPTG-inducible expression plasmid, Cm^R	(5)
pLMG18-lgt^{Ec}	pLMG18 expressing *E. coli lgt*	This study
pLMG18-lgt^{Sa}	pLMG18 expressing *S. aureus lgt*	This study
pLMG18-lgt^{Pa}	pLMG18 expressing *P. aeruginosa lgt*	This study
pLMG18-$lspA^{Ec}$	pLMG18 expressing *E. coli lspA*	This study
pLMG18-lnt^{Ec}	pLMG18 expressing *E. coli lnt*	This study
pLMG18-$lolCDE^{Ec}$	pLMG18 expressing *E. coli lolCDE*	This study
pGFP	pBla_Short encoding sfGFP	(6)
pEX18gm	Suicide cloning vector, Gm^R	(7)

References

1. **Baba T**, **Ara T**, **Hasegawa M**, **Takai Y**, **Okumura Y**, **Baba M**, **Datsenko KA**, **Tomita M**, **Wanner BL**, **Mori H**. 2006. Construction of Escherichia coli K-12 in-frame, single-gene knockout mutants: the Keio collection. Mol Syst Biol **2**:2006.0008.

2. **Ho H**, **Miu A**, **Alexander MK**, **Garcia NK**, **Oh A**, **Zilberleyb I**, **Reichelt M**, **Austin CD**, **Tam C**, **Shriver S**, **Hu H**, **Labadie SS**, **Liang J**, **Wang L**, **Wang J**, **Lu Y**, **Purkey HE**, **Quinn J**, **Franke Y**, **Clark K**, **Beresini MH**, **Tan M-W**, **Sellers BD**, **Maurer T**, **Koehler MFT**, **Wecksler AT**, **Kiefer JR**, **Verma V**, **Xu Y**, **Nishiyama M**, **Payandeh J**, **Koth CM**. 2018. Structural basis for dual-mode inhibition of the ABC transporter MsbA. Nature **557**:196–201.

3. **Datsenko KA**, **Wanner BL**. 2000. One-step inactivation of chromosomal genes in Escherichia coli K-12 using PCR products. Proc Natl Acad Sci USA **97**:6640–6645.

4. **Cherepanov PP**, **Wackernagel W**. 1995. Gene disruption in Escherichia coli: TcR and KmR cassettes with the option of Flp-catalyzed excision of the antibiotic-resistance determinant. Gene **158**:9–14.

5. **Tokunaga M**, **Tokunaga H**, **Wu HC**. 1982. Post-translational modification and processing of Escherichia coli prolipoprotein in vitro. Proc Natl Acad Sci USA **79**:2255–2259.

6. **Storek KM**, **Auerbach MR**, **Shi H**, **Garcia NK**, **Sun D**, **Nickerson NN**, **Vij R**, **Lin Z**, **Chiang N**, **Schneider K**, **Wecksler AT**, **Skippington E**, **Nakamura G**, **Seshasayee D**, **Koerber JT**, **Payandeh J**, **Smith PA**, **Rutherford ST**. 2018. Monoclonal antibody targeting the β-barrel assembly machine of Escherichia coli is bactericidal. Proc Natl Acad Sci USA **115**:3692–3697.

7. **Hmelo LR**, **Borlee BR**, **Almblad H**, **Love ME**, **Randall TE**, **Tseng BS**, **Lin C**, **Irie Y**, **Storek KM**, **Yang JJ**, **Siehnel RJ**, **Howell PL**, **Singh PK**, **Tolker-Nielsen T**, **Parsek MR**, **Schweizer HP**, **Harrison JJ**. 2015. Precision-engineering the Pseudomonas aeruginosa genome with two-step allelic exchange. Nat Protoc **10**:1820–1841.

Table S2: Primers used in this study for strain generation and quantitative PCR

Primer	Sequence (5' to 3')
Strain generation	
CFT073Δ*lgt*.F	TTTCAATCGCTGTTCTCTTTCAGCGAAATAACAAGAACTTGTGGTGACAG GTGTAGGCTGGAGCTGCTTC
CFT073Δ*lgt.R*	CCTTCGTCGAGCACTTTTTGCATCAGTTCTAAATACTGTTTCATGGTTCC CATATGAATA**TCCTCCT**TAGTTCCTATTC
MG1655Δ*lolCDE*.F	CGGGGGCTTTTCAGATTAGCCCTGACGATCACTTACAGTTCAGACGTTTACCCAT CTTGCTTTCGCTTATATACTCGTGTCTTTGCTACAGCAACCAGACGGATTTCGTGT AGGCTGGAGCTGCTTC
MG1655Δ*lolCDE.R*	CCCACTGCAACTGCCGACCGCTATCAAACACGCCAAGCGCAATTTTTGTTCCACC AATATCAAACCCGTAATACATTGCCGCTCCTTGTTTTAATGTACTGCCCATATGA ATA**TCCTCCT**TAGTTCCTATTC
PA14*imp*4213_up-F	CCAGTCACGACGTTGCGGTTCGAACAGCTCGTCG
PA14*imp*4213_up-R	ACGTACGTGACCTCGGCCAGCCAGCG
PA14*imp*4213_dn-F	CCGAGGTCACGTACGTCAACCAACGGG
PA14*imp*4213_dn-R	GGAAACAGCTATGACCCATGTCCGAGGCGAGGAAATTC
pLMG18_GA-F	AAATTGTTATCCGCTCACAATTC
pLMG18_GA-R	CAGCCTGATACAGATTAAATCAGAACGC
Quantitative PCR	
lgt.F	CTCGGTGGACGTATTGGTTATG
lgt.R	TCACCACGATAACGCCAATC
lgt.PRB	**/ *56-FAM* /** ACAATTTCC /ZEN/ CGCAGTTTATGGCCG **/ *3IABkFQ* /**
lspA.F	TCGATCTGGGCAGCAAATAC
lspA.R	CGCTATCGGCAAGGAAACTAA
lspA.PRB	**/ *56-FAM* /** TGCAGATTA /ZEN/ AGCGACGGGAACAGC **/ *3IABkFQ* /**

Underlined sequence denotes the inserted ribosome binding site
